# Supplementary material for: The metabolic stress-activated checkpoint LKB1-MARK3 axis acts as a tumor suppressor in high-grade serous ovarian carcinoma
Source: Commun Biol. 2022 Jan 11;5:39. doi: 10.1038/s42003-021-02992-4 (PMC8752757; doi:10.1038/s42003-021-02992-4)
Supplement: Supplementary file 2 — Supplementary information [file 42003_2021_2992_MOESM2_ESM.pdf]

Supplementary information

The LKB1-MARK3 axis is a novel metabolic stress-activated checkpoint dysregulated in high-grade serous ovarian carcinomas

The file contains

Supplementary Figures 1 – 22

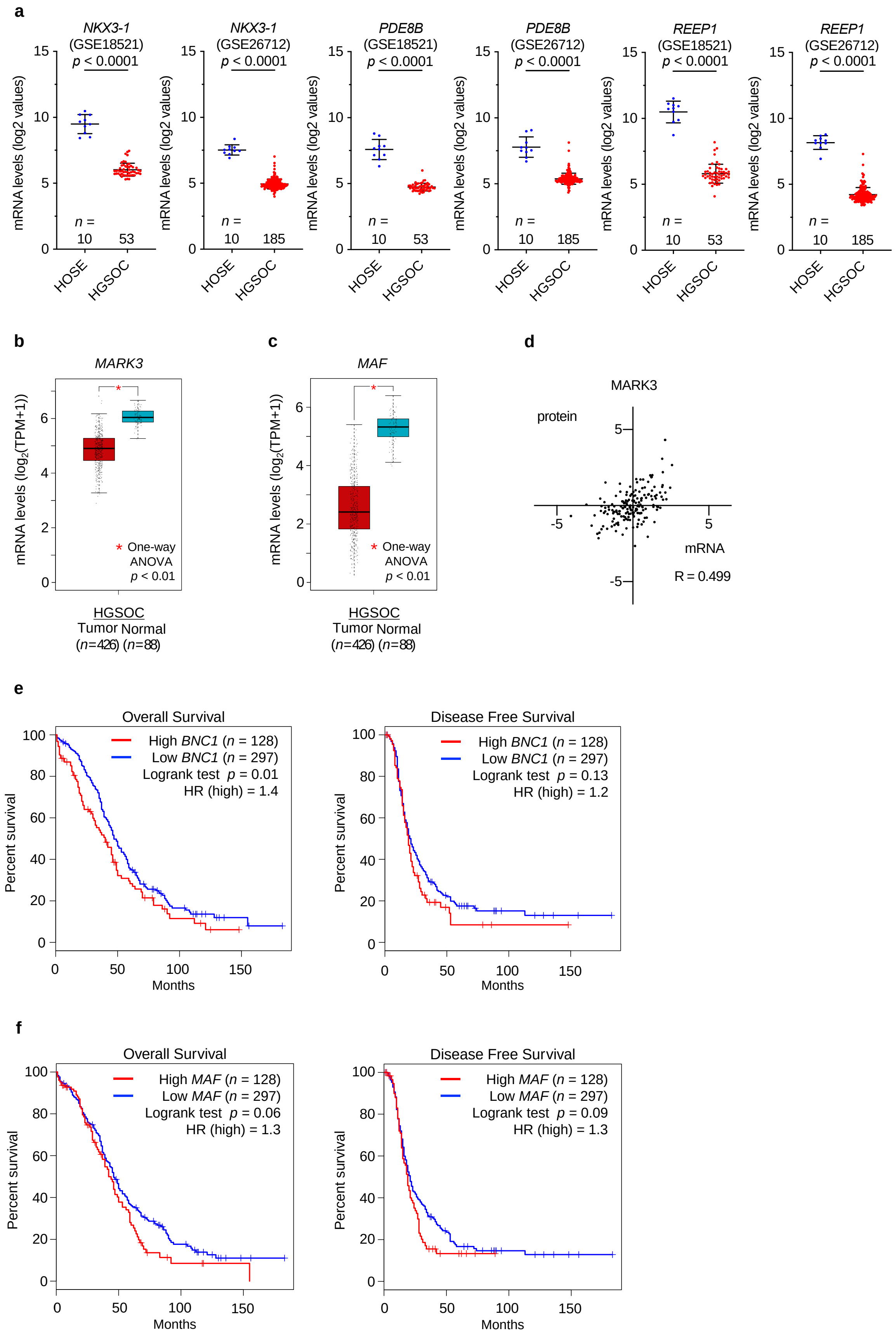
a
NKX3-1 (GSE18521) p < 0.0001
NKX3-1 (GSE26712) p < 0.0001
PDE8B (GSE18521) p < 0.0001
PDE8B (GSE26712) p < 0.0001
REEP1 (GSE18521) p < 0.0001
REEP1 (GSE26712) p < 0.0001
mRNA levels (log2 values)
n = 10 53
n = 10 185
HOSE
HGSOC
b
MARK3
mRNA levels (log2(TPM+1))
* One-way ANOVA p < 0.01
HGSOC
Tumor Normal
(n=426) (n=88)
c
MAF
mRNA levels (log2(TPM+1))
* One-way ANOVA p < 0.01
HGSOC
Tumor Normal
(n=426) (n=88)
d
MARK3
protein
mRNA
R = 0.499
e
Overall Survival
High BNC1 (n = 128)
Low BNC1 (n = 297)
Logrank test p = 0.01
HR (high) = 1.4
Disease Free Survival
High BNC1 (n = 128)
Low BNC1 (n = 297)
Logrank test p = 0.13
HR (high) = 1.2
Percent survival
Months
f
Overall Survival
High MAF (n = 128)
Low MAF (n = 297)
Logrank test p = 0.06
HR (high) = 1.3
Disease Free Survival
High MAF (n = 128)
Low MAF (n = 297)
Logrank test p = 0.09
HR (high) = 1.3
Percent survival
Months

Supplementary Fig. 1 *In silico* analysis identified HGSOC-associated genes. a mRNA expression levels of *NKX3-1*, *PED8B*, and *REEP1* in two independent microarray datasets. GSE18521 and GSE26712 offer microarray data of human ovarian surface epithelial cell (HOSE) samples and HGSOC samples [GSE18521 (HOSE: *n* = 10, HGSOC: *n* = 53) and GSE26712 (HOSE: *n* = 10, HGSOC: *n* = 185)]. Statistical analysis was performed using unpaired Student's t-test. **b** *MARK3* mRNA expression is downregulated in HGSOC. $\log_2$(TPM+1) values of *MARK3* in TCGA and GTEx datasets are shown. Red box and blue box represent cancer tissues and normal counterparts, respectively. TPM: Transcripts per million transcripts. Statistical analysis was performed using one-way ANOVA. **c** *MAF* mRNA expression is downregulated in HGSOC. $\log_2$(TPM+1) values of *MAF* in TCGA and GTEx datasets are shown. Red box and blue box represent cancer tissues and normal counterparts, respectively. Statistical analysis was performed using one-way ANOVA. **d** mRNA expression levels (z-score) of *MARK3* are plotted against protein expression levels of *MARK3* (*n* = 174). The data were analyzed using Pearson's correlation coefficients. **e** Kaplan-Meier survival curves. Kaplan-Meier survival curves classified by high (*n* = 128) or low (*n* = 297) *BNC1* mRNA expression in the TCGA HGSOC cohort. High *BNC1* mRNA expression group exhibits poor overall survival. **f** Kaplan-Meier survival curves. Kaplan-Meier survival curves classified by high (*n* = 128) or low (*n* = 297) *MAF* mRNA expression in the TCGA HGSOC cohort.

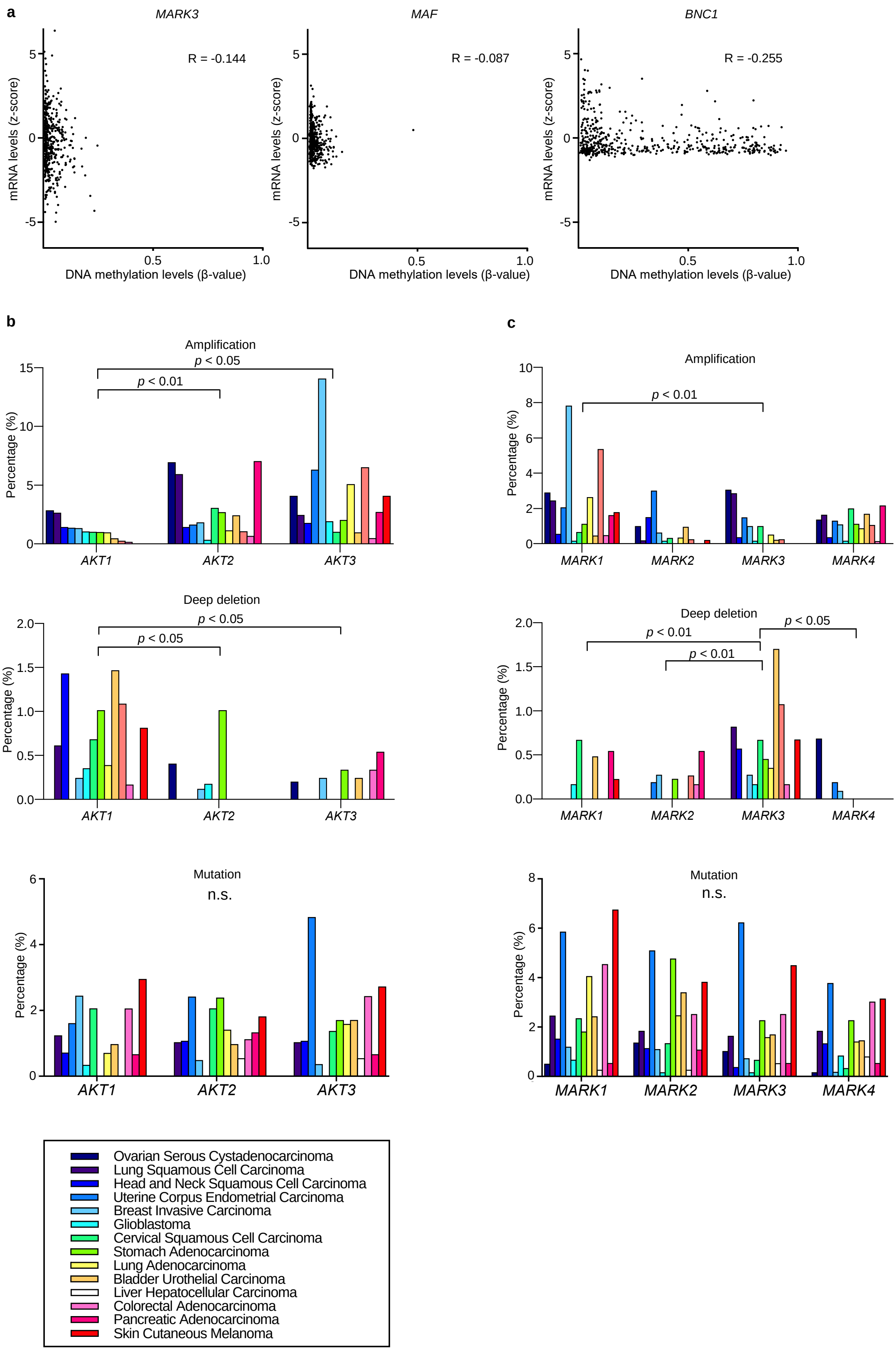
a
MARK3
MAF
BNC1
R = -0.144
R = -0.087
R = -0.255
mRNA levels (z-score)
DNA methylation levels (β-value)
b
c
Amplification
Deep deletion
Mutation
n.s.
p < 0.05
p < 0.01
Percentage (%)
AKT1
AKT2
AKT3
MARK1
MARK2
MARK3
MARK4
Ovarian Serous Cystadenocarcinoma
Lung Squamous Cell Carcinoma
Head and Neck Squamous Cell Carcinoma
Uterine Corpus Endometrial Carcinoma
Breast Invasive Carcinoma
Glioblastoma
Cervical Squamous Cell Carcinoma
Stomach Adenocarcinoma
Lung Adenocarcinoma
Bladder Urothelial Carcinoma
Liver Hepatocellular Carcinoma
Colorectal Adenocarcinoma
Pancreatic Adenocarcinoma
Skin Cutaneous Melanoma

Supplementary Fig. 2 Distinct mechanisms to dysregulate the MARK3 activity. a DNA promoter methylation levels and mRNA expression levels of *MARK3*, *MAF*, and *BNC1* are plotted using the TCGA HGSOC dataset (n = 489). The data were analyzed using Pearson's correlation coefficients. **b** Bar graph shows the population of AKT family mutations among 14 cancer types studied in TCGA. Statistical analysis was performed using unpaired Student's t-test. n.s.: not significant. **c** Bar graph shows the population of MARK family mutations among 14 cancer types studied in TCGA. Statistical analysis was performed using unpaired Student's t-test. n.s.: not significant. The sample sizes are as follows; Ovarian Serous Cystadenocarcinoma (n = 489), Lung Squamous Cell Carcinoma (n = 487), Head and Neck Squamous Cell Carcinoma (n = 279), Uterine Corpus Endometrial Carcinoma (n = 363), Breast Invasive Carcinoma (n = 816), Glioblastoma (n = 563), Cervical Squamous Cell Carcinoma (n = 293), Stomach Adenocarcinoma (n = 295), Lung Adenocarcinoma (n = 511), Bladder Urothelial Carcinoma (n = 408), Liver Hepatocellular Carcinoma (n = 367), Colorectal Adenocarcinoma (n = 592), Pancreatic Adenocarcinoma (n = 184) and Skin Cutaneous Melanoma (n = 367).

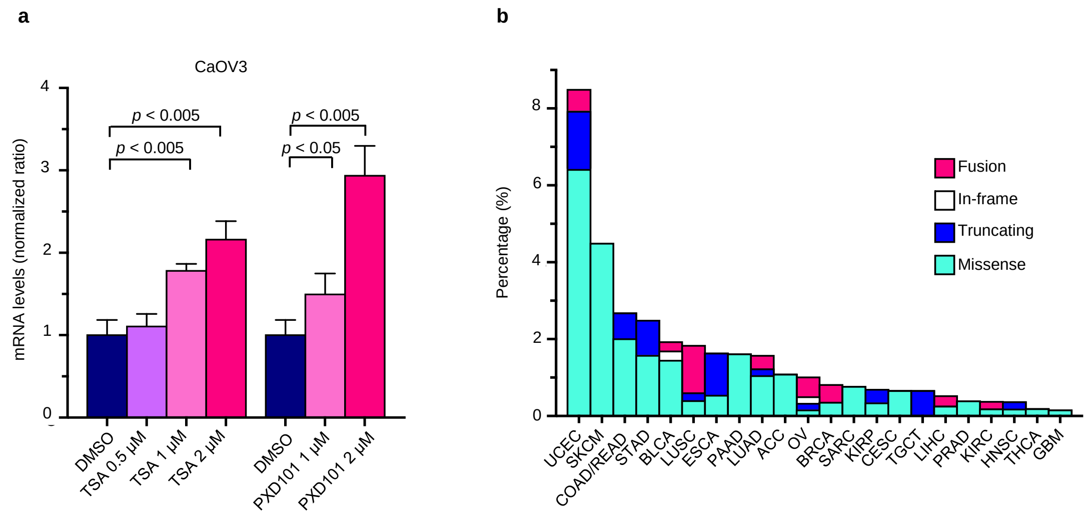

Supplementary Fig. 3 Epigenetic regulation and mutation profile of *MARK3*. a Chemical screening is performed using molecular inhibitors targeting epigenetic regulatory enzymes as follows; DNMT, pan-HDAC, class IIa HDAC, EZH2, G9a, and LSD1. Among them, trichostatin A (TSA) and belinostat (PXD101) upregulate *MARK3* mRNA expression in CaOV3 cells. Error bars represent mean ± standard deviation (SD) of three biological replicates. Statistical analysis was performed using unpaired Student's t-test. **b** *MARK3* mutation profile across 22 cancer types sourced from TCGA. UCEC: Uterine Corpus Endometrial Carcinoma, SKCM: Skin Cutaneous Melanoma, COAD: Colon Adenocarcinoma, READ: Rectum Adenocarcinoma, STAD: Stomach Adenocarcinoma, LUSC: Lung Squamous Cell Carcinoma, ESCA: Esophageal Adenocarcinoma, PAAD: Pancreatic Adenocarcinoma, LUAD: Lung Adenocarcinoma, ACC: Adrenocortical Carcinoma, OV: Ovarian Serous Cystadenocarcinoma, BRCA: Breast Invasive Carcinoma, SARC: Sarcoma, KIRP: Kidney Renal Papillary Cell Carcinoma, CESC: Cervical Squamous Cell Carcinoma, TGCT: Testicular Germ Cell Tumors, LIHC: Liver Hepatocellular Carcinoma, PRAD: Prostate Adenocarcinoma, KIRC: Kidney Renal Clear Cell Carcinoma, HNSC: Head and Neck Squamous Cell Carcinoma, THCA: Thyroid Carcinoma, GBM: Glioblastoma.

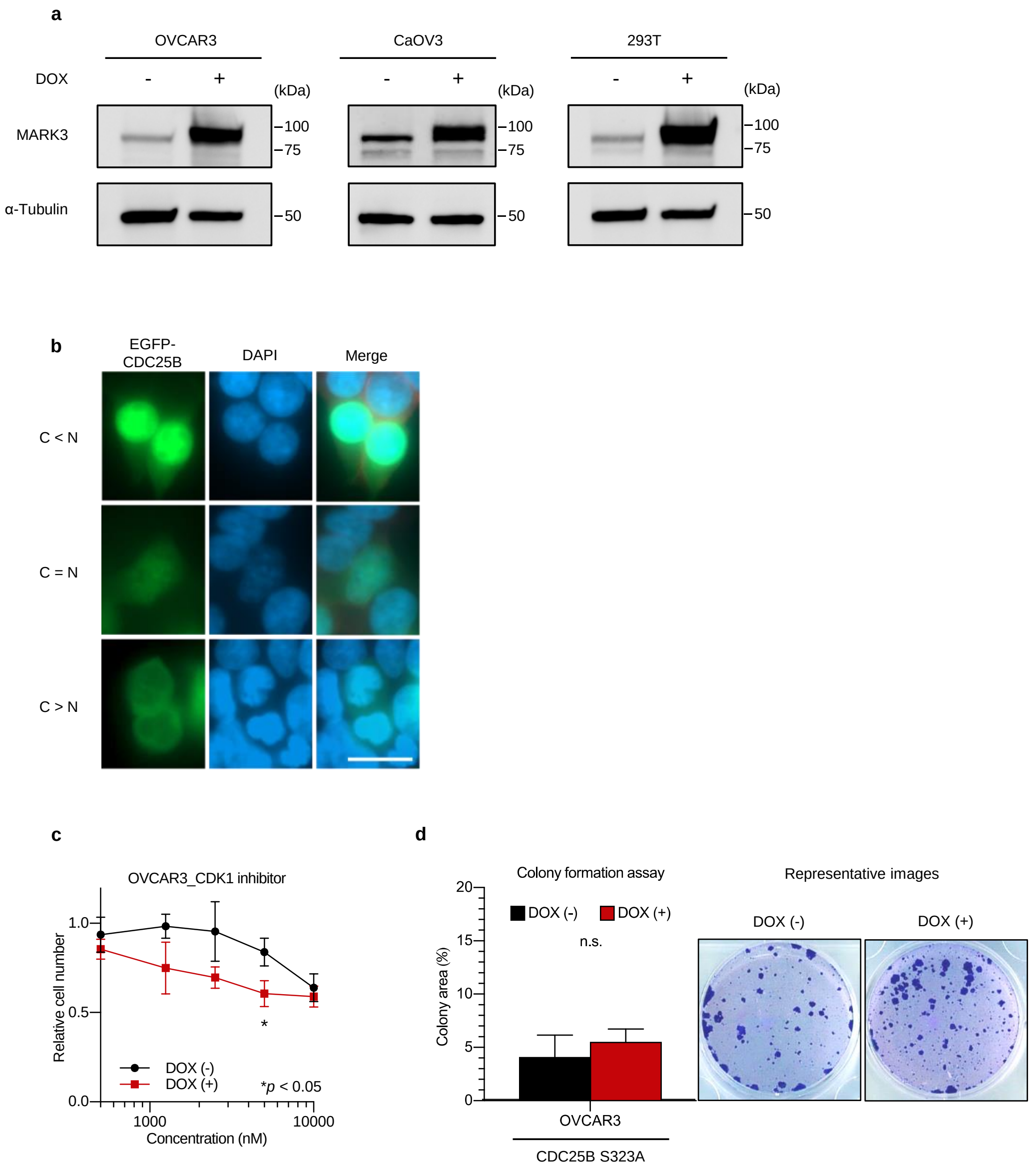

Supplementary Fig. 4 MARK3 is conditionally expressed in various cell lines using doxycycline (DOX)-inducible system. a MARK3 is conditionally expressed upon doxycycline (DOX) treatment (1 μg/ml) for 24 h. **b** Immunocytochemistry shows the representative images to define subcellular localization of EGFP-tagged CDC25B in 293T cells for the following subgroups; C < N: Nuclear-dominant. C = N: Equivalent. C > N: Cytoplasmic-dominant. Scale bars, 20 μm. **c** Cell viability assay for the combination therapy of Ro-3306, a CDK1 inhibitor, and DOX-inducible MARK3 in OVCAR3 cells. The cytotoxic effects of the CDK1 inhibitor is enhanced by MARK3 overexpression. Error bars represent mean ± standard deviation (SD) of three biological replicates. Statistical analysis was performed using unpaired Student's t-test. **d** Colony formation assay in MARK3 DOX-inducible OVCAR3 cells with CDC25B S323A overexpression. Error bars represent mean ± standard deviation (SD) of three biological replicates. Statistical analysis was performed using unpaired Student's t-test.

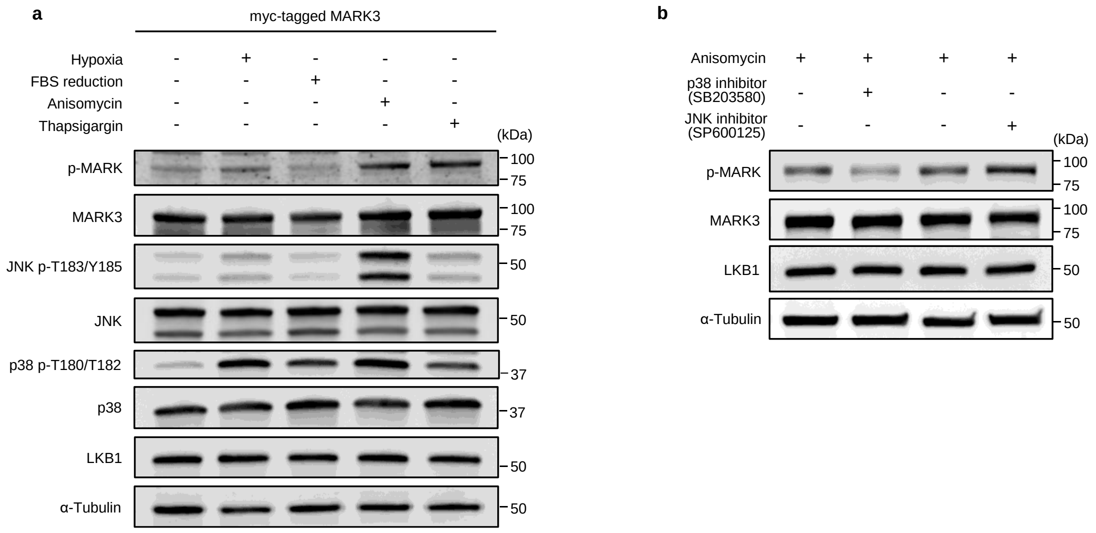

Supplementary Fig. 5 MARK3 is activated by metabolic stress inducers. a immunoblotting of screening and validation for MARK3 activation upon exposure to metabolic stresses. To validate MARK3 activation in non-doxycycline (DOX) system, myc-tagged MARK3 is transiently transfected to 293T cells. Anisomycin and thapsigargin increase the kinase-activated phosphorylation of myc-tagged MARK3. **b** Immunoblotting of inhibition experiments for the upstream regulators of MARK3. p38 inhibition interferes with DOX-inducible MARK3 activation in response to anisomycin treatment.

a

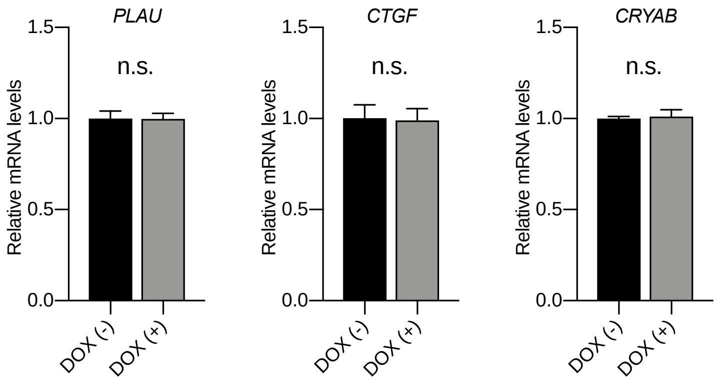

b

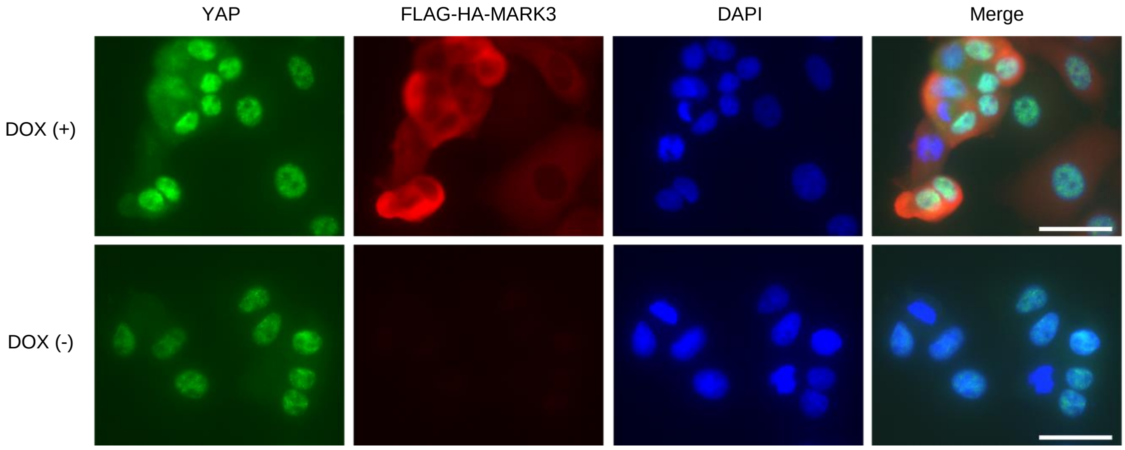

Supplementary Fig. 6 Validation studies for the effect of MARK3 on Hippo signaling genes. **a** *PLAU, CTGF and CRYAB* mRNA expression levels with or without DOX treatment in parental OVCAR3 cells. No significant change is observed for these Hippo target genes in the condition of parental OVCAR3 cells. Error bars represent mean ± standard deviation (SD) of three biological replicates. Statistical analysis was performed using unpaired Student's t-test. **b** Immunocytochemistry shows that YAP subcellular localization with or without DOX treatment in MARK3 DOX-inducible OVCAR3 cells. Anti-HA antibody was used to detect FLAG-HA-MARK3. Scale bars, 40 μm.

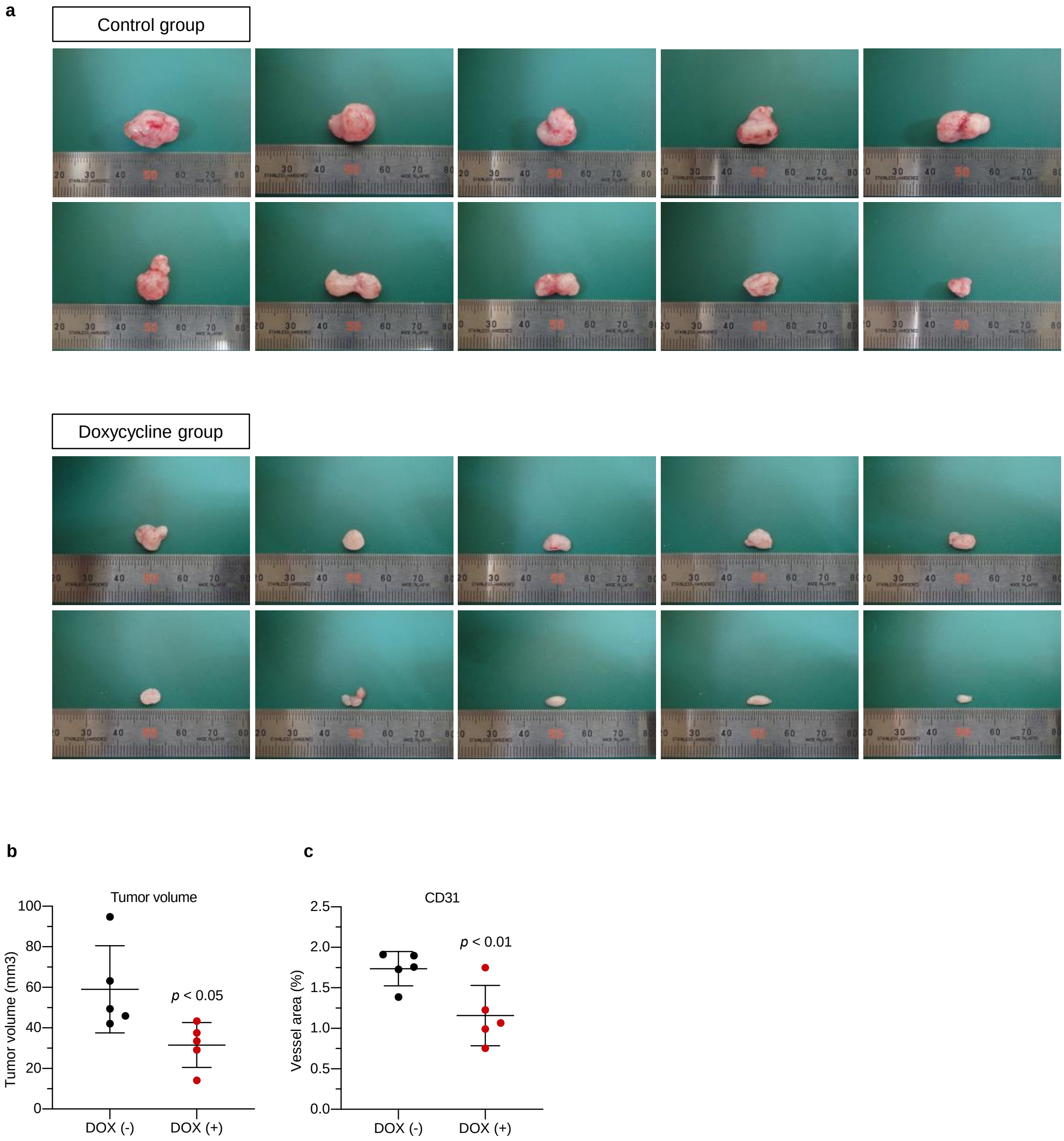

Supplementary Fig. 7 MARK3 inhibits *in vivo* tumor growth of OVCAR3 cells. a Mouse xenograft experiment using MARK3 DOX-inducible OVCAR3 cells. The images of mouse tumors resected at 50 days from treatment. n = 10 in DOX negative group and n = 10 in DOX positive group. **b** The tumor volume distribution of mouse xenografts resected at 30 days from treatment. n = 5 in DOX negative group and n = 5 in DOX positive group. Error bars represent mean ± SD. Statistical analysis was performed using unpaired Student's t-test. **c** CD31 staining area of mouse xenografts resected at 30 days from treatment. n = 5 in DOX negative group and n = 5 in DOX positive group. Error bars represent mean ± SD. Statistical analysis was performed using unpaired Student's t-test.

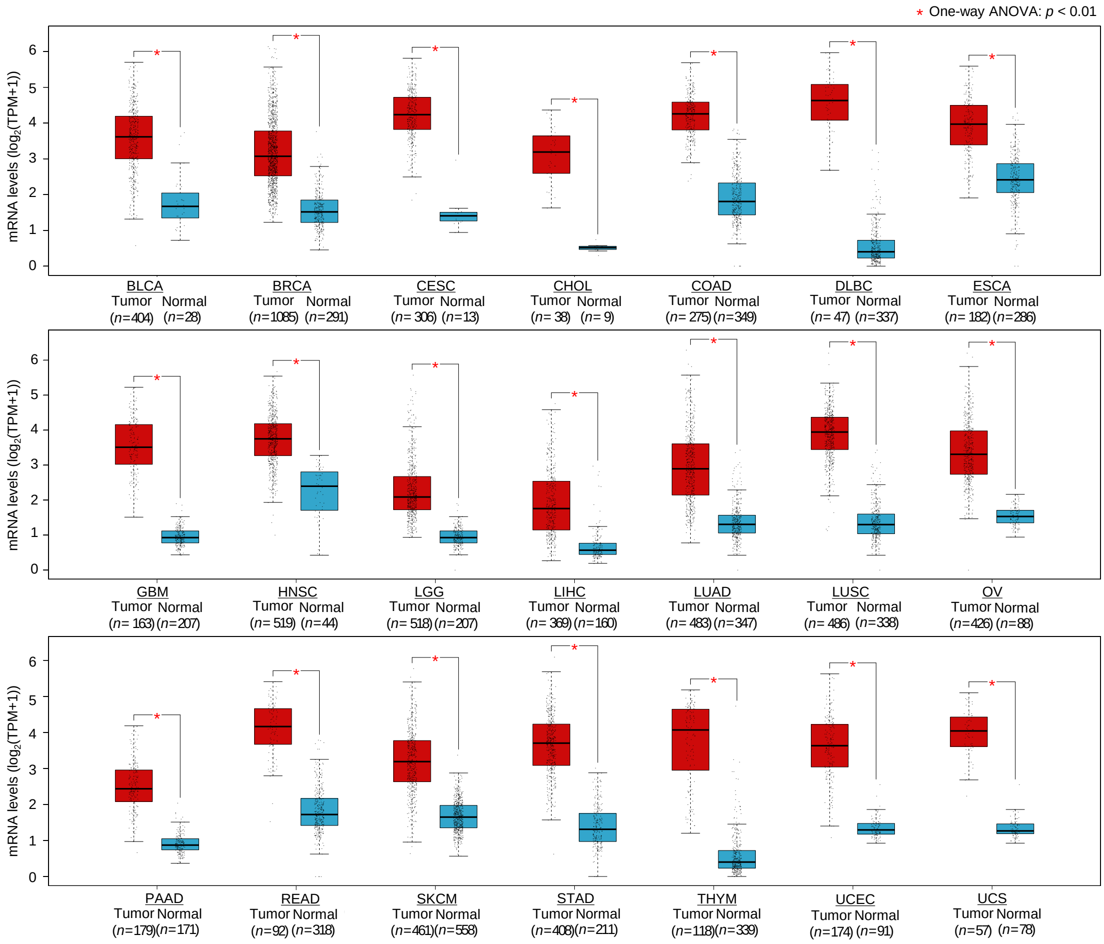

Supplementary Fig. 8 *CHEK1* is upregulated in the majority of cancer types. $\log_2$(TPM+1) values of *CHEK1* in TCGA and GTEx datasets across 21 cancer types are shown. Red box and blue box represent cancer tissues and normal counterparts, respectively. *CHEK1* mRNA expression is upregulated in the majority of cancer types. BLCA: Bladder Urothelial Carcinoma, BRCA: Breast Invasive Carcinoma, CESC: Cervical Squamous Cell Carcinoma, CHOL: Cholangiocarcinoma, COAD: Colon Adenocarcinoma, DLBC: Lymphoid Neoplasm Diffuse Large B-cell Lymphoma, ESCA: Esophageal Carcinoma, GBM: Glioblastoma Multiforme, HNSC: Head and Neck Squamous Cell Carcinoma, LGG: Brain Lower Grade Glioma, LIHC: Liver Hepatocellular Carcinoma, LUAD: Lung Adenocarcinoma, LUSC: Lung Squamous Cell Carcinoma, OV: Ovarian Serous Cystadenocarcinoma, PRAD: Prostate Adenocarcinoma, READ: Rectum Adenocarcinoma, SKCM: Skin Cutaneous Melanoma, STAD: Stomach Adenocarcinoma, THYM: Thymoma, UCEC: Uterine Corpus Endometrial Carcinoma, UCS: Uterine Carcinosarcoma. Statistical analysis was performed using one-way ANOVA.

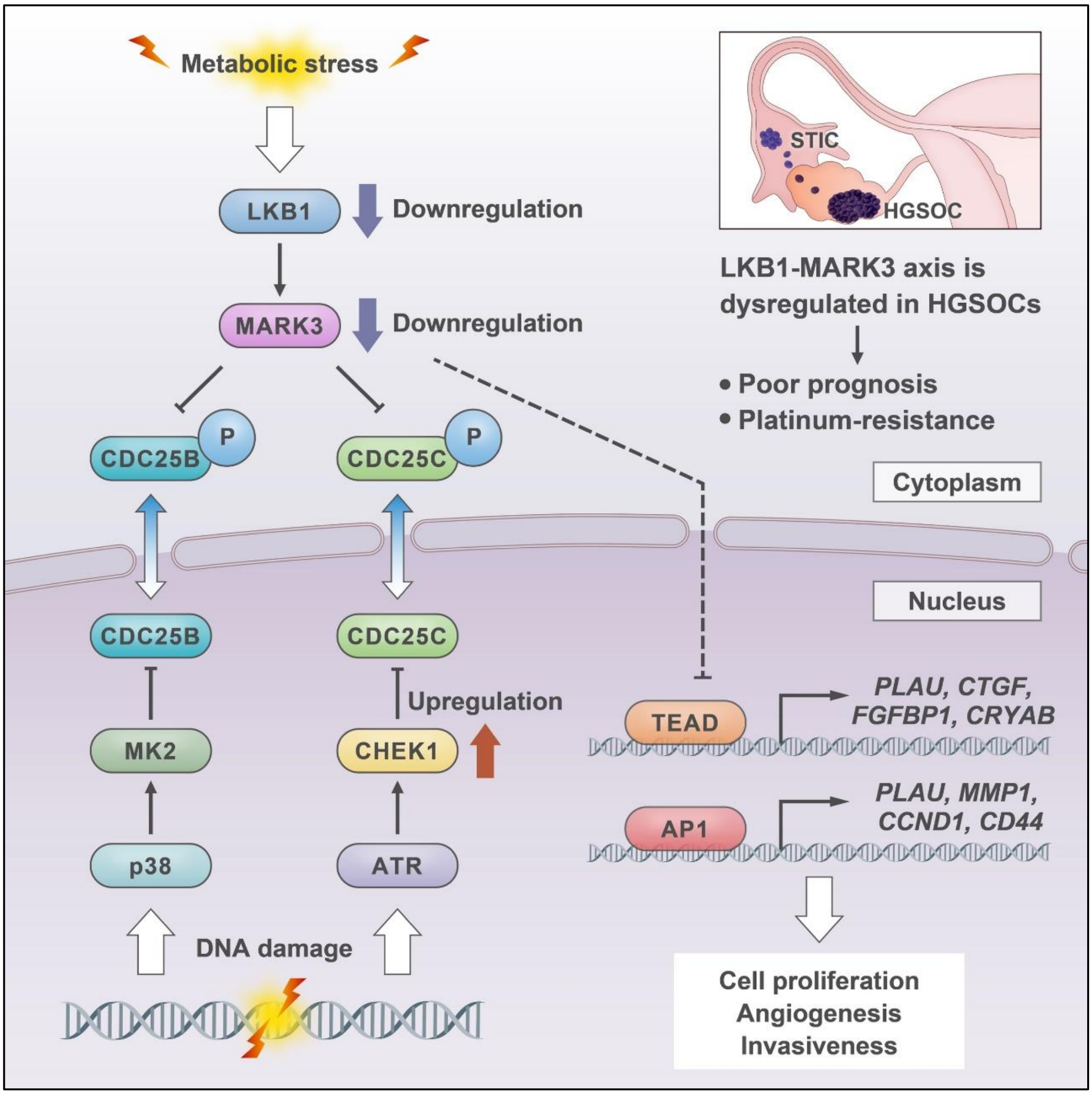

Supplementary Fig. 9 Schematic of the signal transduction systems around the LKB1-MARK3 axis. The cytoplasmic checkpoint LKB1-MARK3 axis is activated by metabolic stress, such as protein synthesis inhibition and unfolded protein response, whereas the nuclear checkpoints, like the ATR-CHEK1 axis and the p38-MAPKAPK2 axis, are activated by DNA damage. In chromosomally unstable cancer cells, *CHEK1* is upregulated to maintain genomic integrity. In contrast, *LKB1* and *MARK3* are downregulated in HGSOCs seemingly to undermine metabolic stress-activated G2/M phase checkpoint. The LKB1-MARK3 axis represses cell cycle progression and angiogenesis by antagonizing nuclear translocation of CDC25B and CDC25C and the transcription activities of TEAD and AP-1 complex.

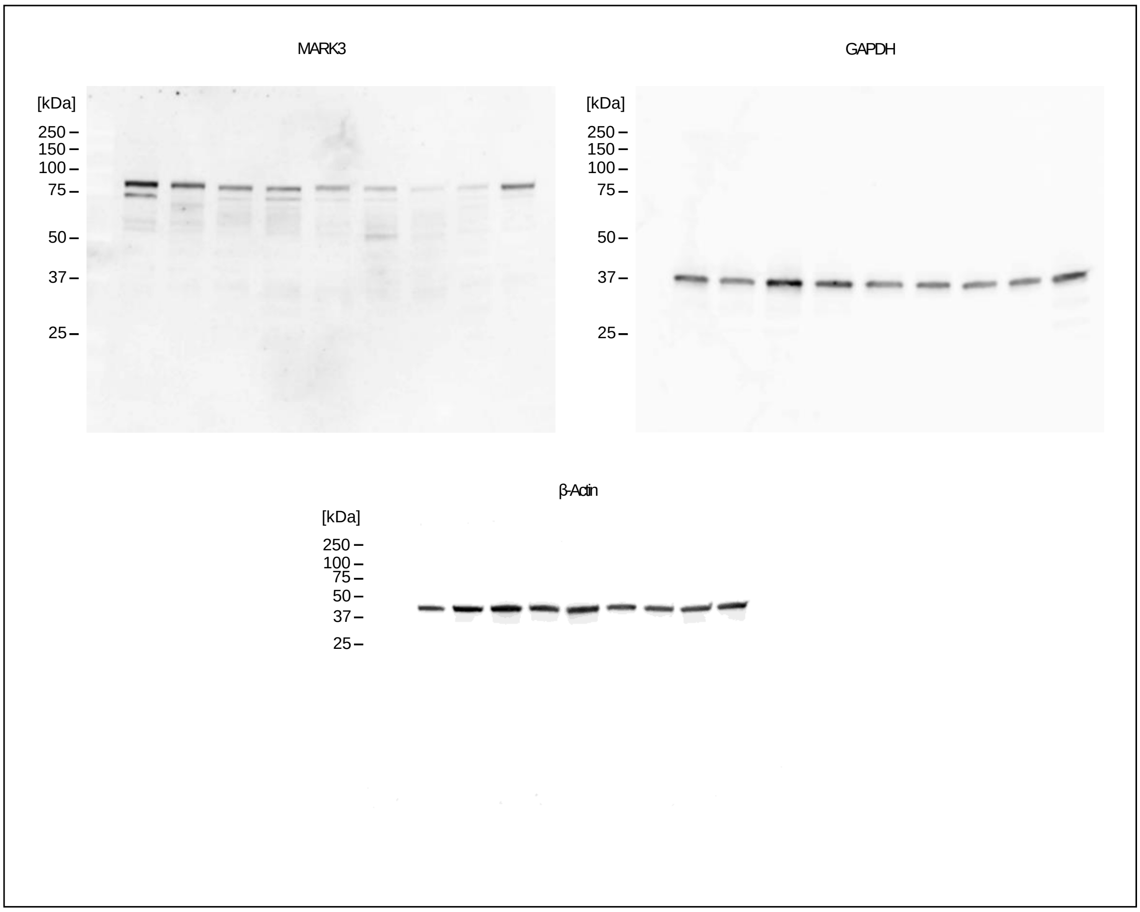

Supplementary Fig. 10 Uncropped images. Membranes used for immunodetection shown in Figure 1i.

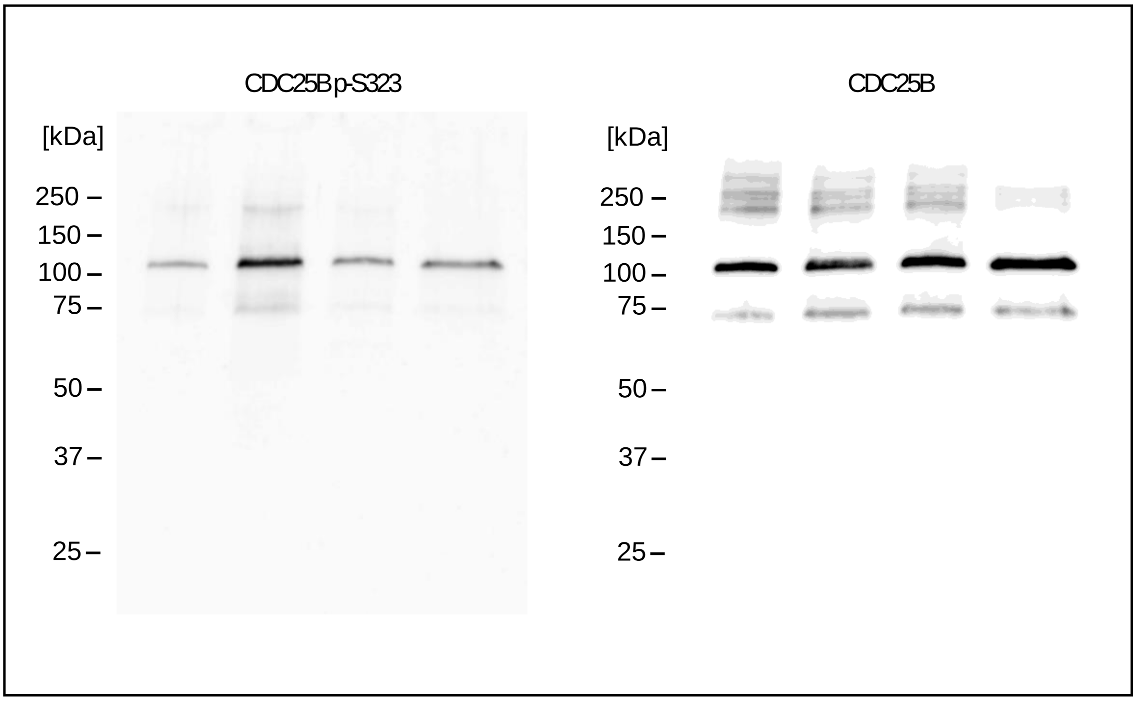

Supplementary Fig. 11 Uncropped images. Membranes used for immunodetection shown in Figure 4b.

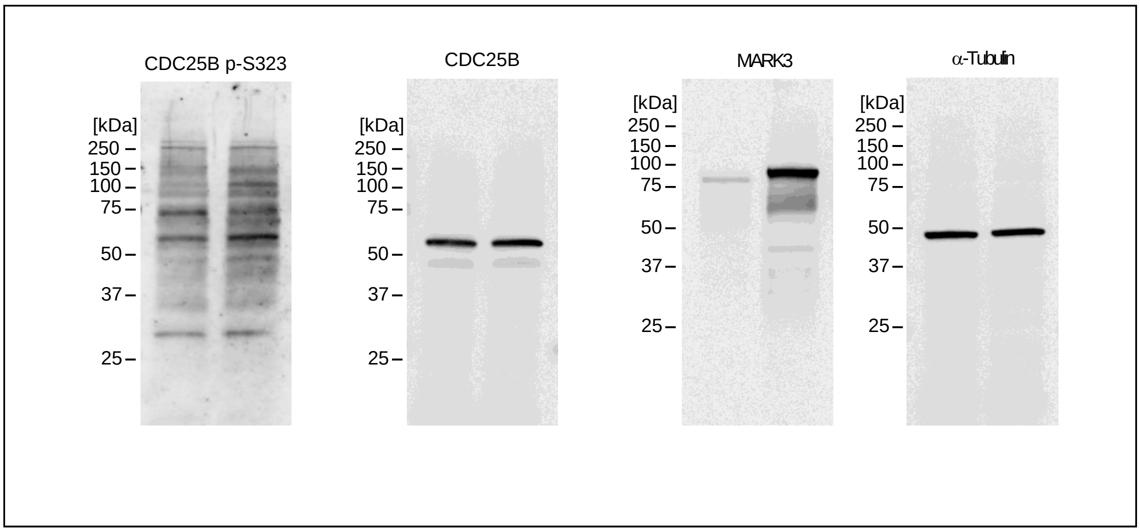

Supplementary Fig. 12 Uncropped images. Membranes used for immunodetection shown in Figure 4c.

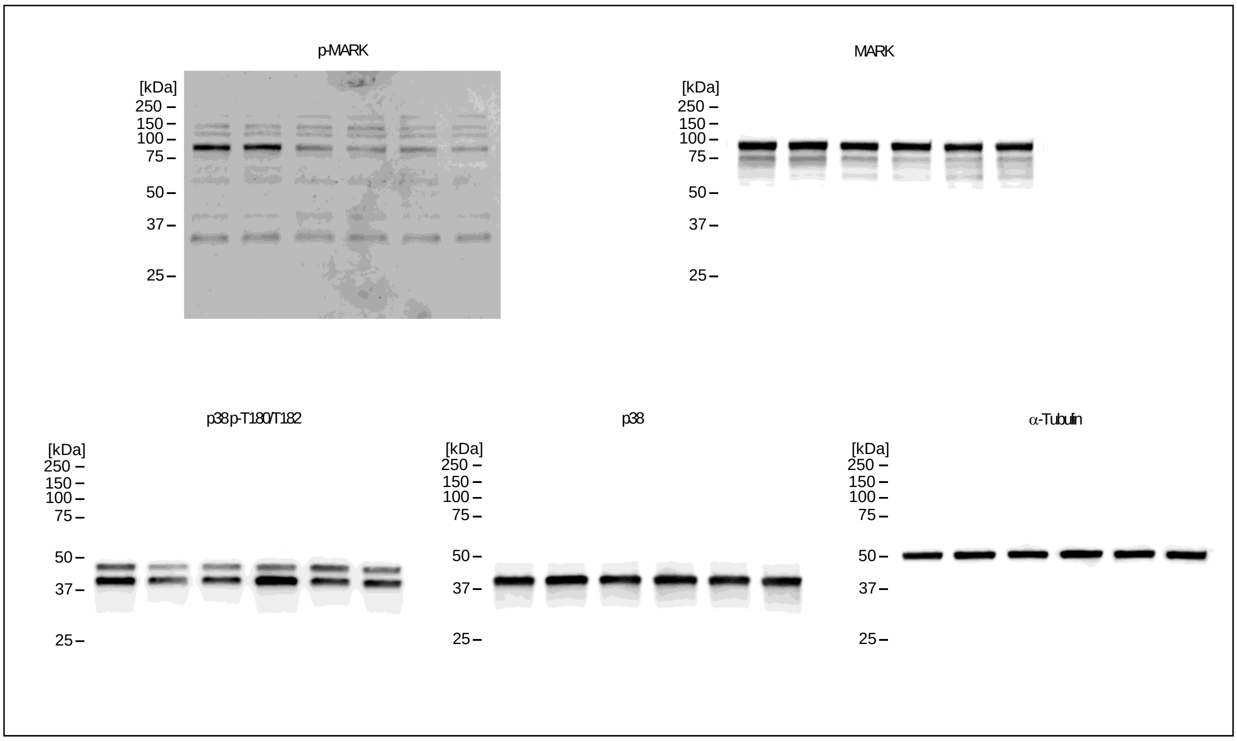

Supplementary Fig. 13 Uncropped images. Membranes used for immunodetection shown in Figure 5a.

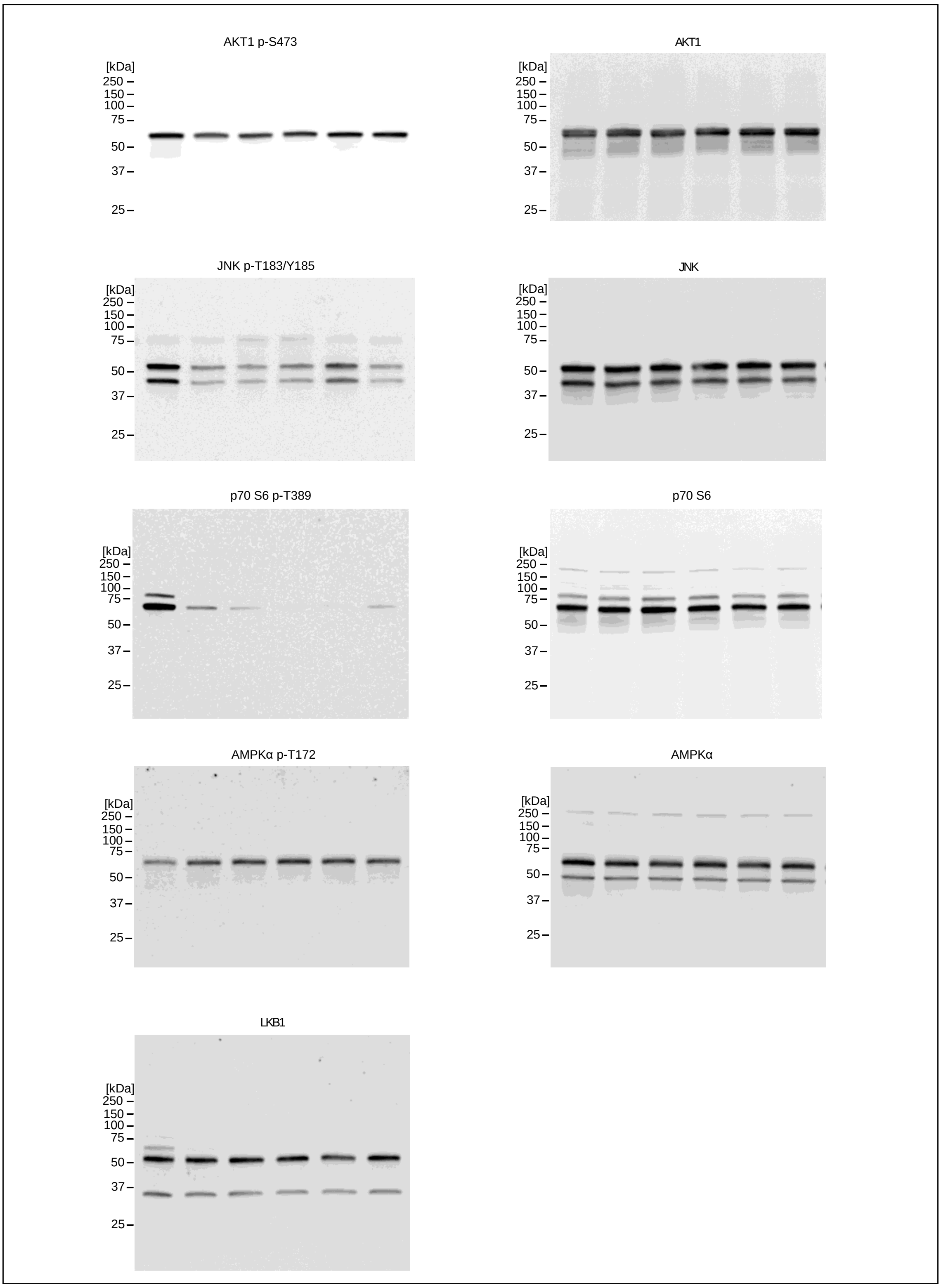

Supplementary Fig. 14 Uncropped images. Membranes used for immunodetection shown in the remaining Figure 5a.

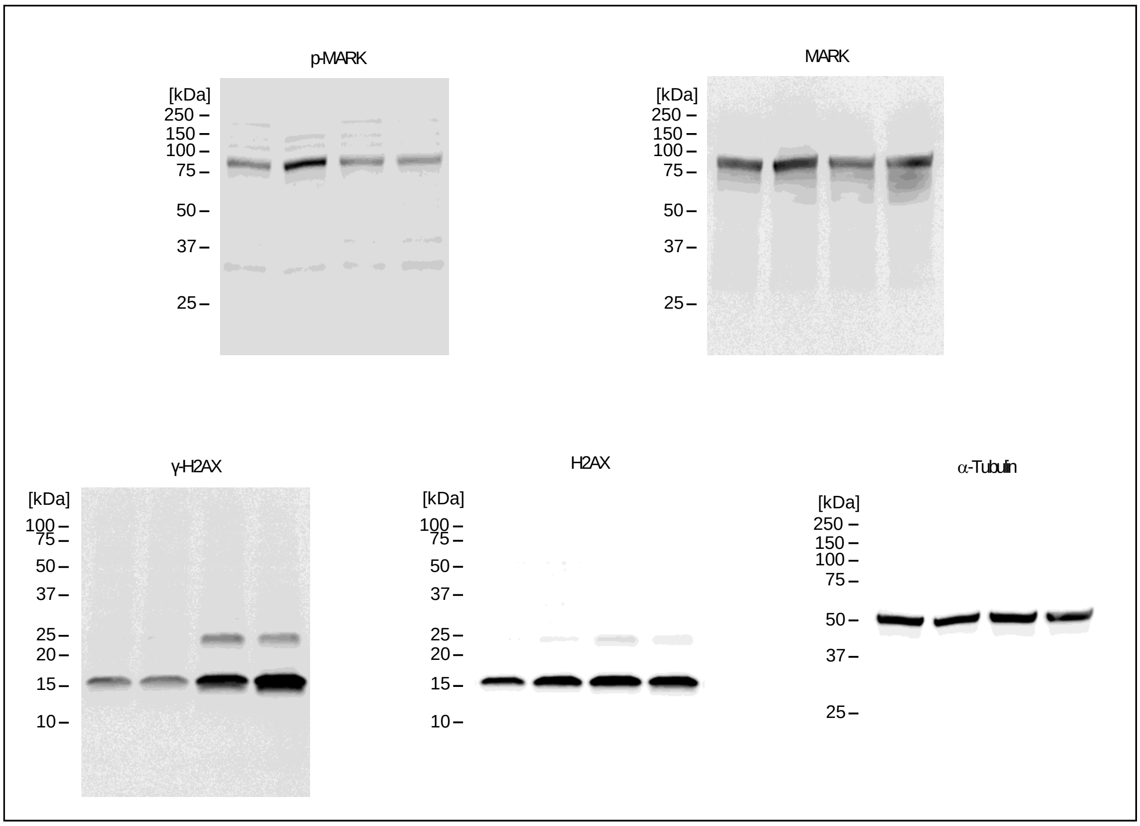

Supplementary Fig. 15 Uncropped images. Membranes used for immunodetection shown in Figure 5b.

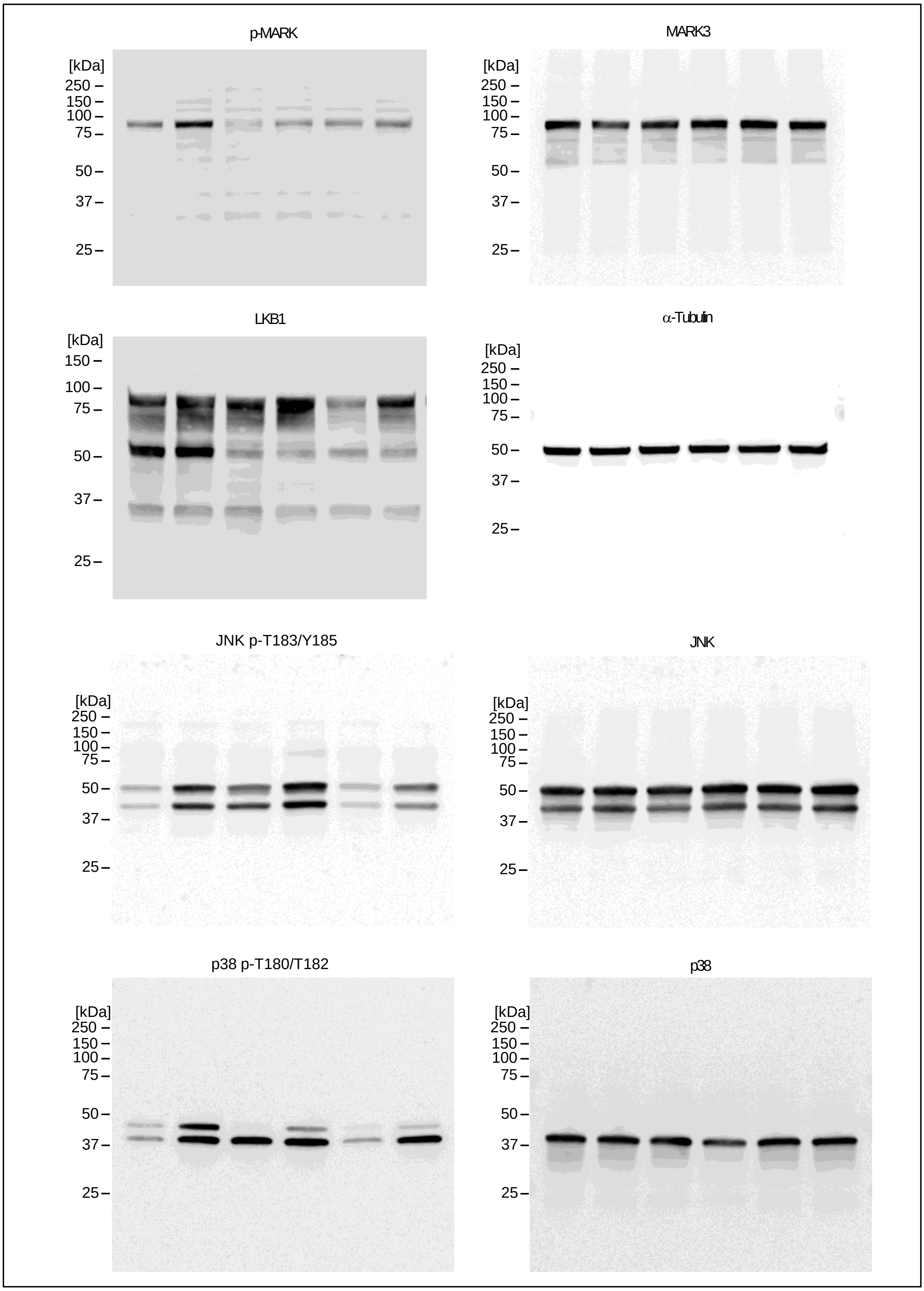

Supplementary Fig. 16 Uncropped images. Membranes used for immunodetection shown in Figure 5c.

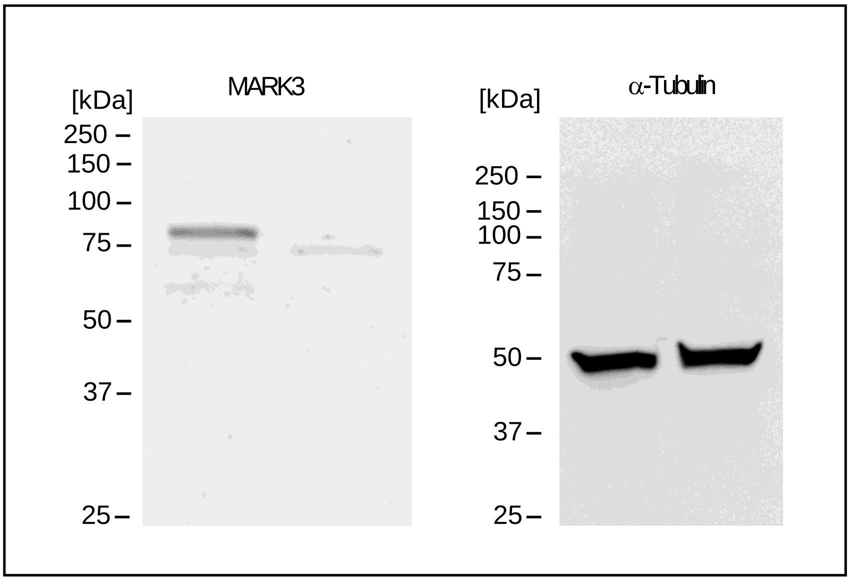

Supplementary Fig. 17 Uncropped images. Membranes used for immunodetection shown in Figure 5d.

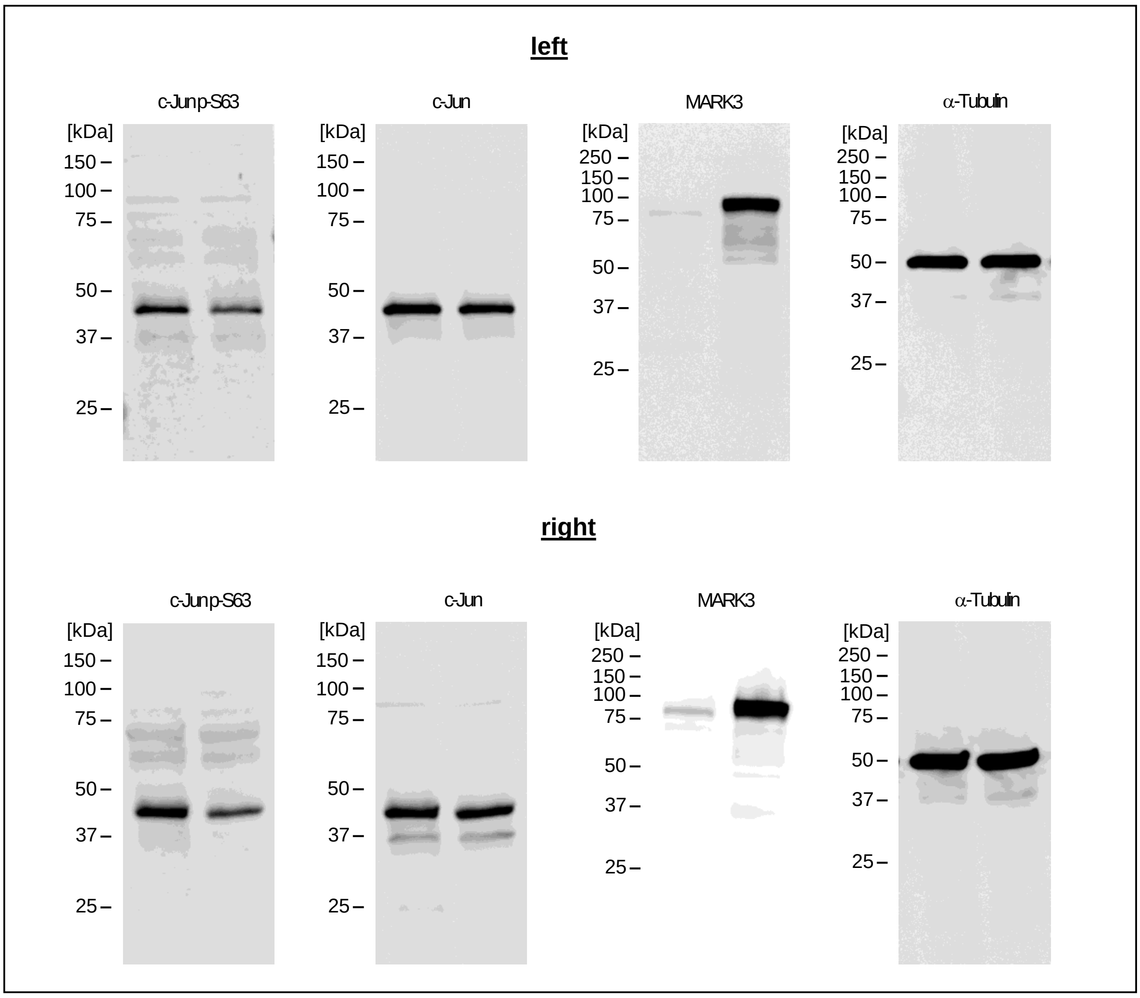

Supplementary Fig. 18 Uncropped images. Membranes used for immunodetection shown in Figure 6c.

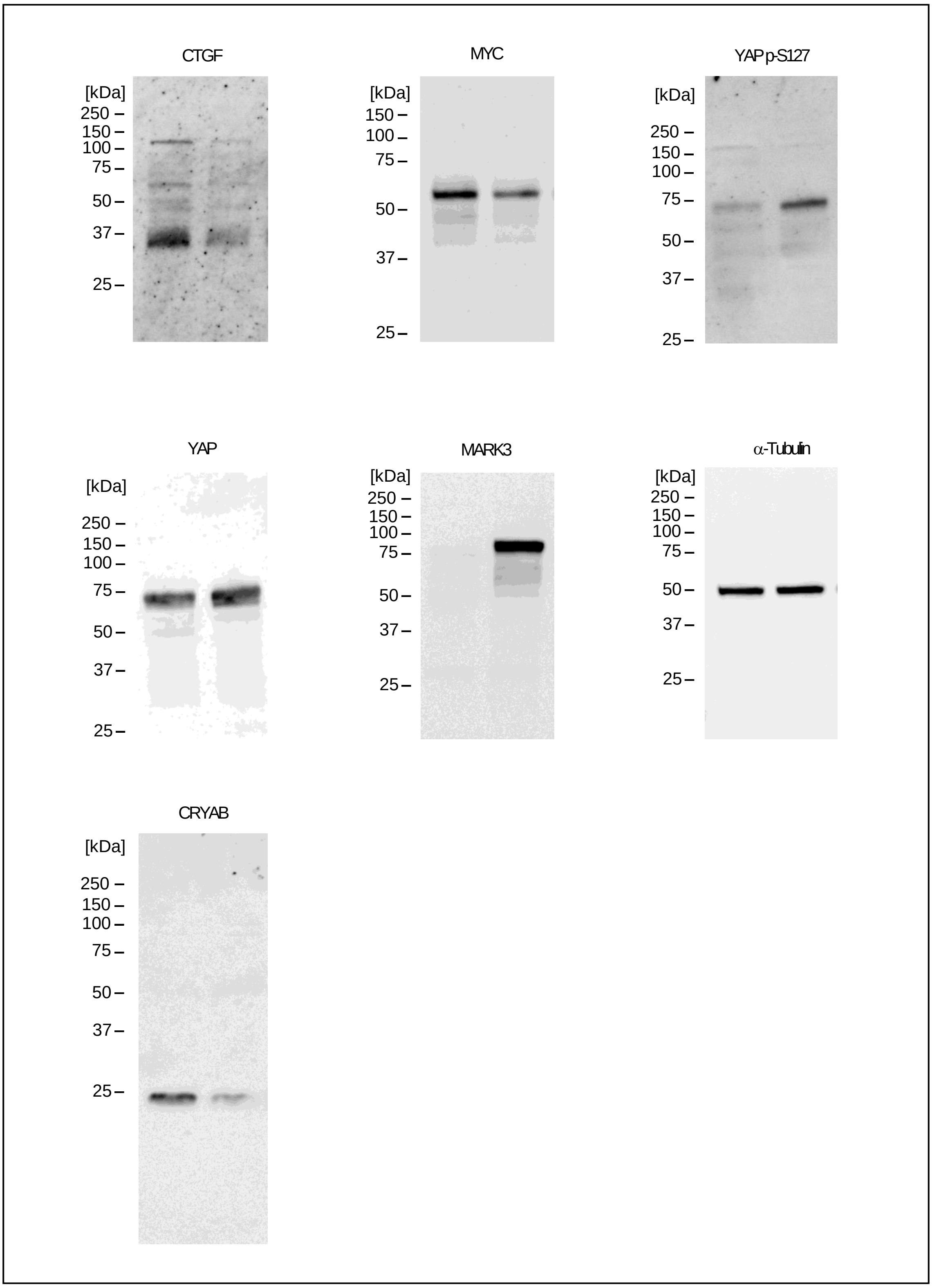

Supplementary Fig. 19 Uncropped images. Membranes used for immunodetection shown in Figure 6g.

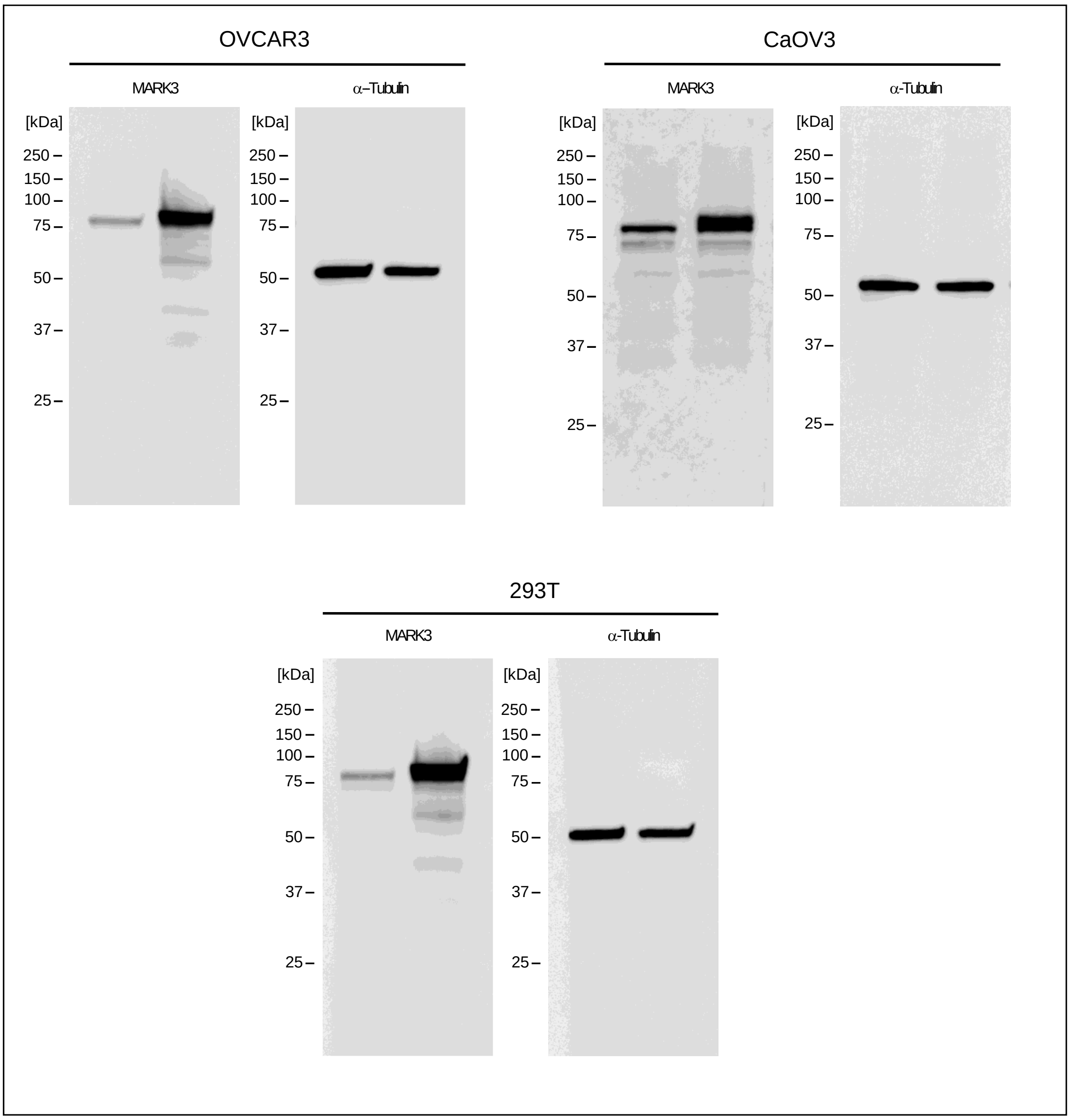

Supplementary Fig. 20 Uncropped images. Membranes used for immunodetection shown in Supplementary Figure 4.

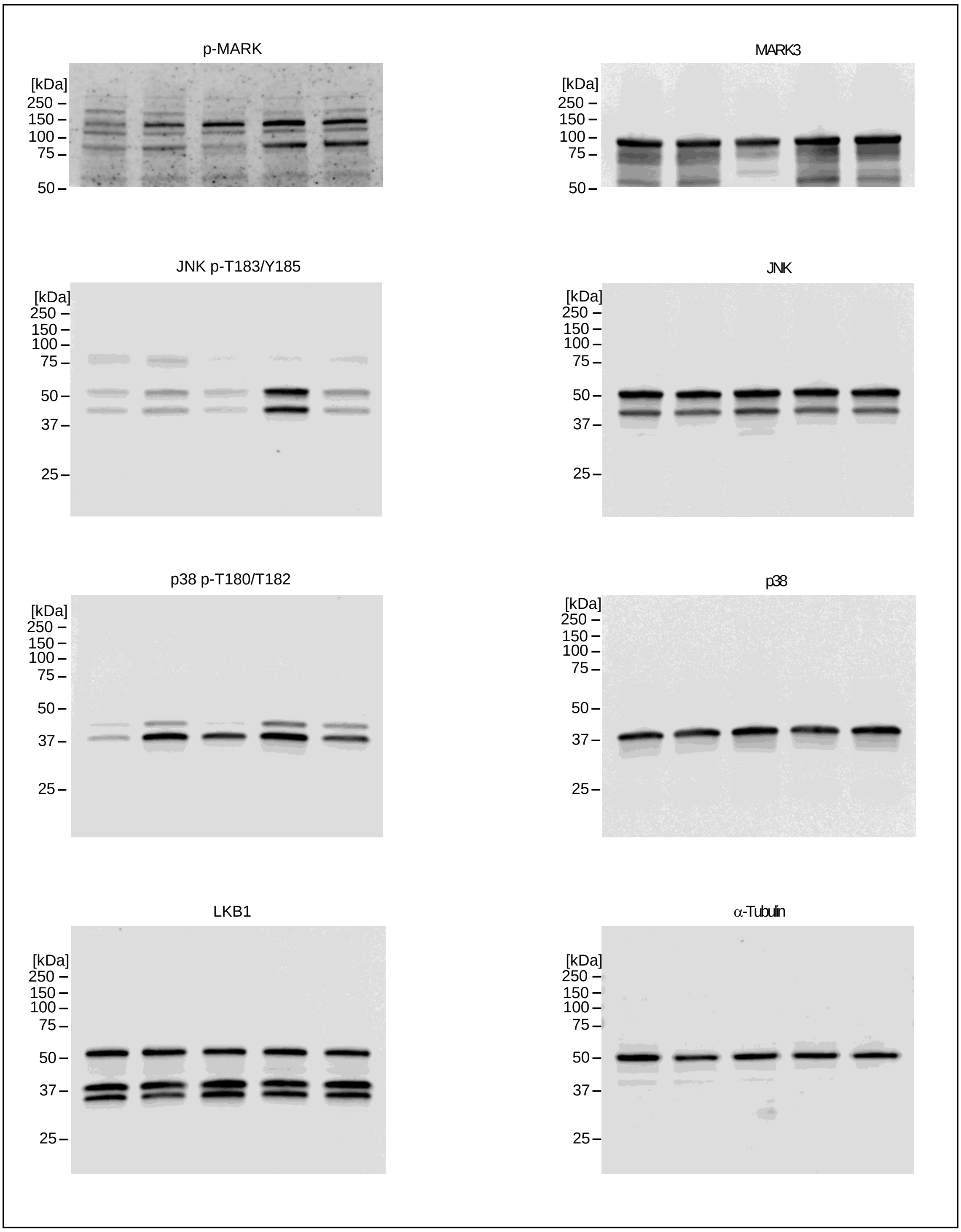

Supplementary Fig. 21 Uncropped images. Membranes used for immunodetection shown in Supplementary Figure 5a.

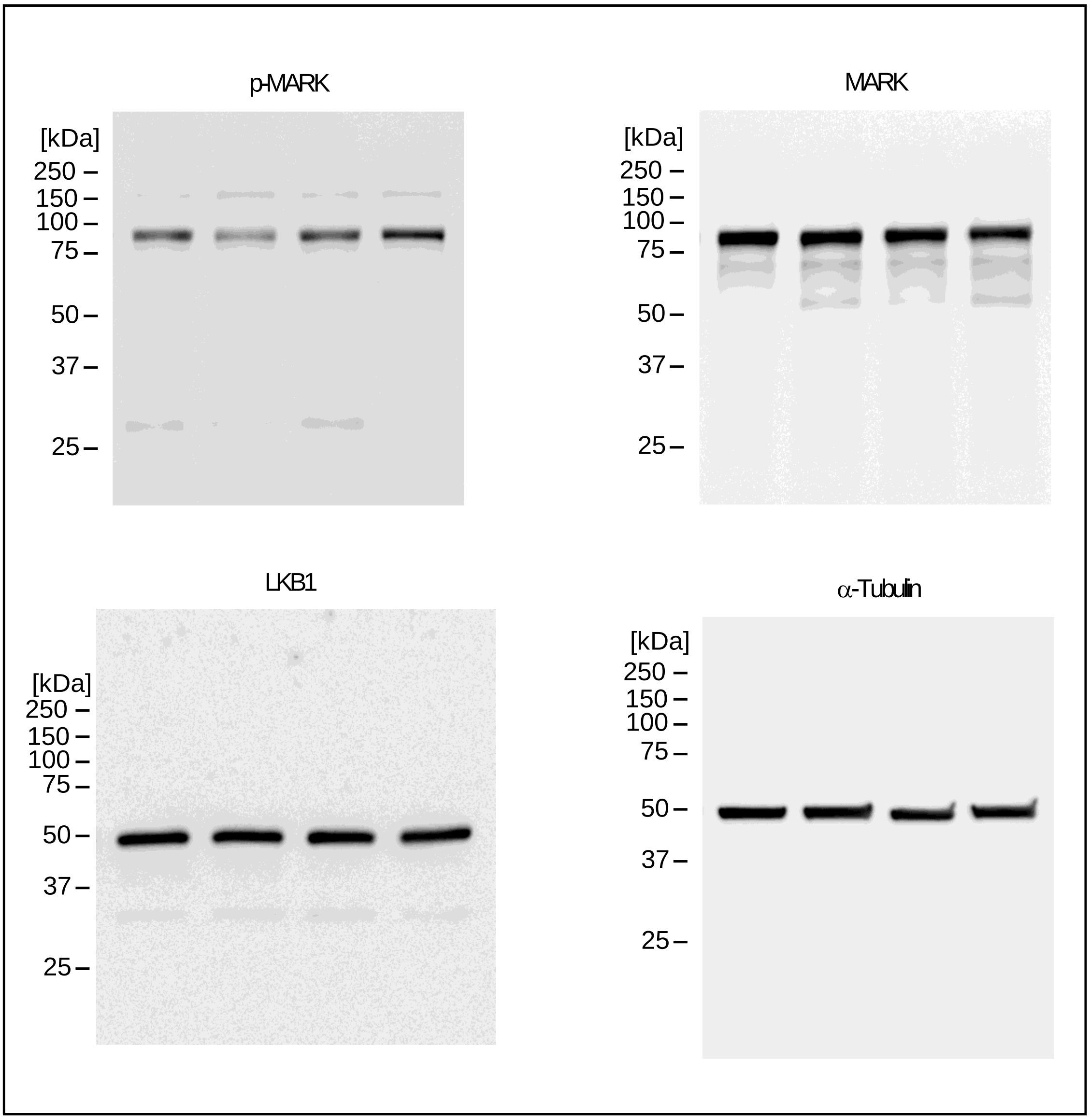

Supplementary Fig. 22 Uncropped images. Membranes used for immunodetection shown in Supplementary Figure 5b.
