# Supplementary material for: The metabolic stress-activated checkpoint LKB1-MARK3 axis acts as a tumor suppressor in high-grade serous ovarian carcinoma
Source: Commun Biol. 2022 Jan 11;5:39. doi: 10.1038/s42003-021-02992-4 (PMC8752757; doi:10.1038/s42003-021-02992-4)
Supplement: Supplementary file 3 — Description of Additional Supplementary Files [file 42003_2021_2992_MOESM3_ESM.pdf]

Description of Additional Supplementary Files

File name: Supplementary Data 1.
Description: MARK3 overexpression exerts tumor-suppressive effects in OVCAR3 cells. Pathway analysis using IPA indicated that overexpression of MARK3 would decrease the activity of oncogenes such as MYC, ESR1, KRAS, and VEGF, and increase the activity of tumor suppressor genes such as PTEN, CDKN1A, and CDKN2A.

File name: Supplementary Data 2.
Description: The results of the TF motif enrichment analysis (OVCAR3). ATAC-seq was performed on samples of OVCAR3 cells overexpressing MARK3, and a peak versus motif matrix was generated using HOMER by combining ATAC-seq peaks and JASPAR core non-redundant position frequency matrices on vertebrates. We also integrated a peak versus motif matrix and a peak versus intensity matrix into the significance of TF motif enrichment matrix using the Module Map algorithm of Genomica.

File name: Supplementary Data 3.
Description: The results of the TF motif enrichment analysis (293T). ATAC-seq was performed on samples of 293T cells overexpressing MARK3, and a peak versus motif matrix was generated using HOMER by combining ATAC-seq peaks and JASPAR core non-redundant position frequency matrices on vertebrates. We also integrated a peak versus motif matrix and a peak versus intensity matrix into the significance of TF motif enrichment matrix using the Module Map algorithm of Genomica.

File name: Supplementary Data 4.
Description: Information of certified cell lines. All cell lines were certified by STR profiling cell line authentication.

File name: Supplementary Data 5.
Description: Information of primers. Details of the primer sequences used in this study are shown.

File name: Supplementary Data 6.
Description: The raw data used to generate the figures in this study. Detailed values for individual data are shown.

File name: Supplementary Data 7.
Description: The raw data and t-test results of the mouse xenograft experiments. Detailed values for individual data are shown.
