# Supplementary material for: The metabolic stress-activated checkpoint LKB1-MARK3 axis acts as a tumor suppressor in high-grade serous ovarian carcinoma
Source: Commun Biol. 2022 Jan 11;5:39. doi: 10.1038/s42003-021-02992-4 (PMC8752757; doi:10.1038/s42003-021-02992-4)
Supplement: Supplementary file 11 — Reporting Summary [file 42003_2021_2992_MOESM11_ESM.pdf]

nature portfolio

Corresponding author(s): Ryuji Hamamoto and Syuzo Kaneko

Last updated by author(s): Nov 28, 2021

Reporting Summary

Nature Portfolio wishes to improve the reproducibility of the work that we publish. This form provides structure for consistency and transparency in reporting. For further information on Nature Portfolio policies, see our Editorial Policies and the Editorial Policy Checklist.

Statistics

For all statistical analyses, confirm that the following items are present in the figure legend, table legend, main text, or Methods section.

n/a	Confirmed	
☐	☒	The exact sample size (*n*) for each experimental group/condition, given as a discrete number and unit of measurement
☐	☒	A statement on whether measurements were taken from distinct samples or whether the same sample was measured repeatedly
☐	☒	The statistical test(s) used AND whether they are one- or two-sided<br>*Only common tests should be described solely by name; describe more complex techniques in the Methods section.*
☐	☒	A description of all covariates tested
☐	☒	A description of any assumptions or corrections, such as tests of normality and adjustment for multiple comparisons
☐	☒	A full description of the statistical parameters including central tendency (e.g. means) or other basic estimates (e.g. regression coefficient) AND variation (e.g. standard deviation) or associated estimates of uncertainty (e.g. confidence intervals)
☐	☒	For null hypothesis testing, the test statistic (e.g. *F*, *t*, *r*) with confidence intervals, effect sizes, degrees of freedom and *P* value noted<br>*Give P values as exact values whenever suitable.*
☒	☐	For Bayesian analysis, information on the choice of priors and Markov chain Monte Carlo settings
☒	☐	For hierarchical and complex designs, identification of the appropriate level for tests and full reporting of outcomes
☐	☒	Estimates of effect sizes (e.g. Cohen's *d*, Pearson's *r*), indicating how they were calculated

Our web collection on statistics for biologists contains articles on many of the points above.

Software and code

Policy information about availability of computer code

Data collection	Two independent datasets (GSE18521 and GSE26712), containing microarray data of HOSE and HGSOC samples, were obtained from Gene Expression Omnibus (GEO). Gene expression levels were fitted to a log2 scale. RNA-seq, DNA promoter methylation, DNA copy number, gene mutation, and clinical data of HGSOC patients in The Cancer Genome Atlas (TCGA) cohort were sourced from the cBioPortal for Cancer Genomics.
Data analysis	Differential expression analysis between cancer and normal tissues across multiple cancer types was performed by gene expression profiling interactive analysis (GEPIA), in which RNA-seq data from TCGA and the Genotype-Tissue Expression (GTEx) projects were processed. RNA-seq reads were aligned to the human reference genome NCBI build hg38 using STAR. Transcripts per million transcripts (TPM) were calculated using RSEM, and DEGs were extracted using edgeR, in which FDR less than 0.05 were considered statistically significant. KEGG pathway analysis and GO analysis were processed on DAVID (v.6.8). Upstream regulator analysis was performed by ingenuity pathway analysis (IPA). ATAC-seq was performed by Active Motif. FASTQ files were processed for adapter sequence trimming, mapping to hg19 using bowtie2 using the option of -very sensitive -X 2000 and PCR duplicate removal. Mapping quality was assessed by DROMPA. Peaks were called by MACS2 using the options -f BAM -g hs -q 0.01 --nomodel --shift -75 --extsize 150 -B and further filtered with P-value < 10*-10. Peak raw counts were quantile normalized. Transcription factor (TF) motif enrichment analysis was performed as follows. A peak versus motif matrix was generated using HOMER, combining ATAC-seq peaks and JASPAR core non-redundant position frequency matrices on vertebrates. A peak versus motif matrix and a peak versus intensity matrix were integrated into the significance of TF motif enrichment matrix by the Module Map algorithm of Genomica.

For manuscripts utilizing custom algorithms or software that are central to the research but not yet described in published literature, software must be made available to editors and reviewers. We strongly encourage code deposition in a community repository (e.g. GitHub). See the Nature Portfolio guidelines for submitting code & software for further information.

Data

Policy information about availability of data

All manuscripts must include a data availability statement. This statement should provide the following information, where applicable:

- Accession codes, unique identifiers, or web links for publicly available datasets
- A description of any restrictions on data availability
- For clinical datasets or third party data, please ensure that the statement adheres to our policy

Quantitation of blots in figures is provided in Supplementary Data 6. Untrimmed blots for immunoblotting are present in Supplementary Information. Sequence data generated in this study are available at the DNA Data Bank of Japan (DDBJ) (Accession number: DRA010685)

Field-specific reporting

Please select the one below that is the best fit for your research. If you are not sure, read the appropriate sections before making your selection.

[x] Life sciences   [ ] Behavioural & social sciences   [ ] Ecological, evolutionary & environmental sciences

For a reference copy of the document with all sections, see nature.com/documents/nr-reporting-summary-flat.pdf

Life sciences study design

All studies must disclose on these points even when the disclosure is negative.

Sample size	The sample size design was determined based on "significance level", "power", and "effect size". In this study, the statistical significance level was set at less than 0.05 in principle.
Data exclusions	No data were excluded from analyses.
Replication	Each experiment was repeated at least three times, and the experiments throughout the manuscript were successfully reproduced.
Randomization	This study was analyzed based on pure biochemistry and molecular biology and is not a clinical trial. For this reason, we have determined that there is no need for blinding.
Blinding	Similar to the above, this study was analyzed based on pure biochemistry and molecular biology and is not a clinical trial. For this reason, we have determined that there is no need for blinding.

Reporting for specific materials, systems and methods

We require information from authors about some types of materials, experimental systems and methods used in many studies. Here, indicate whether each material, system or method listed is relevant to your study. If you are not sure if a list item applies to your research, read the appropriate section before selecting a response.

Materials & experimental systems

n/a	Involved in the study	
[ ]	[x]	Antibodies
[ ]	[x]	Eukaryotic cell lines
[x]	[ ]	Palaeontology and archaeology
[ ]	[x]	Animals and other organisms
[x]	[ ]	Human research participants
[x]	[ ]	Clinical data
[x]	[ ]	Dual use research of concern

Methods

n/a	Involved in the study	
[x]	[ ]	ChIP-seq
[x]	[ ]	Flow cytometry
[x]	[ ]	MRI-based neuroimaging

Antibodies

Antibodies used	Anti-α-Tubulin mouse monoclonal antibody (CP06 [EMD Millipore]; dilution used in WB: 1:1000); anti-β-Actin rabbit polyclonal antibody (#4967 [Cell Signaling Technology, Danvers, MA, USA]; dilution used in WB: 1:1000); anti-AKT rabbit monoclonal antibody (#4691 [Cell Signaling Technology]; dilution used in WB: 1:1000); anti-phospho-AKT (Ser473) rabbit monoclonal antibody (#4060 [Cell Signaling Technology]; dilution used in WB: 1:2000); anti-AMPKα rabbit polyclonal antibody (#2532 [Cell Signaling Technology]; dilution used in WB: 1:1000); anti-phospho-AMPKα (Thr172) rabbit monoclonal antibody (#2535 [Cell Signaling Technology]; dilution used in WB: 1:1000); anti-CD31 rat antibody (553370 [BD Biosciences]; dilution used in IHC: 1:500); anti-CDC25B rabbit monoclonal antibody (ab124819 [Abcam, Cambridge, UK]; dilution used in WB: 1:1000); anti-phospho-CDC25B (Ser323) rabbit polyclonal antibody (ab53103 [Abcam]; dilution used in WB: 1:300); anti-c-JUN rabbit monoclonal antibody (#9165 [Cell Signaling Technology]; dilution used in WB: 1:1000); anti-phospho-c-JUN (Ser63) rabbit polyclonal antibody (#9261 [Cell Signaling Technology]; dilution used

in WB: 1:1000); anti-CRYAB rabbit monoclonal antibody (#45844 [Cell Signaling Technology]; dilution used in WB: 1:1000); anti-CTGF rabbit monoclonal antibody (#86641 [Cell Signaling Technology]; dilution used in WB: 1:1000); anti-GAPDH rabbit monoclonal antibody (#2118 [Cell Signaling Technology]; dilution used in WB: 1:1000); anti-HA mouse monoclonal antibody (901501 [BioLegend, San Diego, CA, USA]; dilution used in ICC: 1:1000 and in WB: 1:1000); anti-JNK rabbit polyclonal antibody (#9252 [Cell Signaling Technology]; dilution used in WB: 1:1000); anti-phospho-JNK (Thr183/Tyr185) rabbit monoclonal antibody (#4668 [Cell Signaling Technology]; dilution used in WB: 1:1000); anti-LKB1 rabbit monoclonal antibody (#3050 [Cell Signaling Technology]; dilution used in WB: 1:1000); anti-LKB1 rabbit monoclonal antibody (IHC Formulated) (#13031 [Cell Signaling Technology]; dilution used in IHC: 1:250); anti-MARK3 rabbit polyclonal antibody (#9311 [Cell Signaling Technology]; dilution used in WB: 1:1000); anti-MARK3 rabbit polyclonal antibody (ab133708 [Abcam]; dilution used in IHC: 1:100); anti-phospho-MARK family rabbit polyclonal antibody (#4836 [Cell Signaling Technology]; dilution used in WB: 1:1000); anti-p38 rabbit monoclonal antibody (#8690 [Cell Signaling Technology]; dilution used in WB: 1:1000); anti-phospho-p38 (Thr180/Tyr182) rabbit monoclonal antibody (#4511 [Cell Signaling Technology]; dilution used in WB: 1:1000); anti-p53 rabbit monoclonal antibody (#2527 [Cell Signaling Technology]; dilution used in IHC: 1:160); anti-p70 S6 rabbit monoclonal antibody (#2708 [Cell Signaling Technology]; dilution used in WB: 1:1000); anti-phospho-p70 S6 (Thr389) rabbit monoclonal antibody (#9234 [Cell Signaling Technology]; dilution used in WB: 1:1000); anti-PAX8 rabbit polyclonal antibody (10336-1-AP [Proteintech, Rosemont, IL, USA]; dilution used in IHC: 1:1000); anti-YAP rabbit monoclonal antibody (#14074 [Cell Signaling Technology]; dilution used in ICC: 1:100 and in WB: 1:1000); and anti-phospho-YAP (Ser127) family rabbit polyclonal antibody (#13008 [Cell Signaling Technology]; dilution used in WB: 1:1000). The following secondary antibodies were used: Anti-mouse IgG, horseradish peroxidase (HRP)-linked species-specific whole antibody (from sheep) (NA931 [GE Healthcare]; dilution used in WB: 1:5000); anti-rabbit IgG, HRP-linked species-specific whole antibody (from donkey) (NA934 [GE Healthcare]; dilution used in WB: 1:5000); anti-mouse IgG (H+L) Alexa Fluor 488 (from donkey) (A21202 [Thermo Fisher Scientific]; dilution used in ICC: 1:10000); EnVision+ System- HRP Labelled Polymer Anti-Rabbit (K4003 [Agilent Dako], an undiluted solution used in IHC); and biotinylated anti-rat IgG (H+L) (BA-9400 [VECTOR LABLATORIES], dilution used in IHC: 1:100).

Validation

Anti-α-Tubulin mouse monoclonal antibody (CP06 [EMD Millipore]: https://www.citeab.com/antibodies/225659-cp06-anti-alpha-tubulin-mouse-mab-dm1a, anti-β-Actin rabbit polyclonal antibody (#4967 [Cell Signaling Technology, Danvers, MA, USA]): https://www.cellsignal.jp/products/primary-antibodies/b-actin-antibody/4967, anti-AKT rabbit monoclonal antibody (#4691 [Cell Signaling Technology]): https://www.cellsignal.jp/products/primary-antibodies/akt-pan-c67e7-rabbit-mab/4691, anti-phospho-AKT (Ser473) rabbit monoclonal antibody (#4060 [Cell Signaling Technology]): https://www.cellsignal.jp/products/primary-antibodies/phospho-akt-ser473-d9e-xp-rabbit-mab/4060, anti-AMPKα rabbit polyclonal antibody (#2532 [Cell Signaling Technology]): https://www.cellsignal.jp/products/primary-antibodies/ampka-antibody/2532, anti-CD31 rat antibody (553370 [BD Biosciences]): https://www.citeab.com/antibodies/2408223-553370-bd-pharmingen-purified-rat-anti-mouse-cd31, anti-CDC25B rabbit monoclonal antibody (ab124819 [Abcam, Cambridge, UK]): https://www.abcam.com/cdc25b-antibody-epr34592-ab124819.html, anti-phospho-CDC25B (Ser323) rabbit polyclonal antibody (ab53103 [Abcam]): https://www.citeab.com/antibodies/721964-ab53103-anti-cdc25b-phospho-s323-antibody, anti-c-JUN rabbit monoclonal antibody (#9165 [Cell Signaling Technology]): https://www.cellsignal.jp/products/primary-antibodies/c-jun-60a8-rabbit-mab/9165, anti-phospho-c-JUN (Ser63) rabbit polyclonal antibody (#9261 [Cell Signaling Technology]): https://www.cellsignal.jp/products/primary-antibodies/phospho-c-jun-ser63-ii-antibody/9261, anti-CRYAB rabbit monoclonal antibody (#45844 [Cell Signaling Technology]): https://www.cellsignal.jp/products/primary-antibodies/cryab-d6s9e-rabbit-mab/45844, anti-CTGF rabbit monoclonal antibody (#86641 [Cell Signaling Technology]): https://www.cellsignal.jp/products/primary-antibodies/ctgf-d8z8u-rabbit-mab/86641, anti-GAPDH rabbit monoclonal antibody (#2118 [Cell Signaling Technology]): https://www.cellsignal.jp/products/primary-antibodies/gapdh-14c10-rabbit-mab/2118, anti-HA mouse monoclonal antibody (901501 [BioLegend, San Diego, CA, USA]): https://www.biolegend.com/en-us/global-elements/pdf-popup/purified-anti-ha-11-epitope-tag-antibody-11374?filename=Purified%20anti-HA11%20Epitope%20Tag%20Antibody.pdf&pdfgen=true, anti-JNK rabbit polyclonal antibody (#9252 [Cell Signaling Technology]): https://www.cellsignal.jp/products/primary-antibodies/sapk-jnk-antibody/9252, anti-phospho-JNK (Thr183/Tyr185) rabbit monoclonal antibody (#4668 [Cell Signaling Technology]): https://www.cellsignal.jp/products/primary-antibodies/phospho-sapk-jnk-thr183-tyr185-81e11-rabbit-mab/4668, anti-LKB1 rabbit monoclonal antibody (#3050 [Cell Signaling Technology]): https://www.cellsignal.jp/products/primary-antibodies/lkb1-27d10-rabbit-mab/3050, anti-LKB1 rabbit monoclonal antibody (IHC Formulated) (#13031 [Cell Signaling Technology]): https://www.cellsignal.jp/products/primary-antibodies/lkb1-d60c5f10-rabbit-mab-ihc-formulated/13031, anti-MARK3 rabbit polyclonal antibody (#9311 [Cell Signaling Technology]): https://www.cellsignal.jp/products/primary-antibodies/mark3-antibody/9311?site-search-type=Products&utm_app=app&utm_campaign=cep&utm_content=20-cep-97405&utm_conv=mon&utm_medium=digital&utm_region=hq&utm_research=cel&utm_seg=aca&utm_source=blog&utm_stage=ase&utm_strategy=dif&utm_tactic=blg&Ns=product.currentLot.numberOfApplications%7C1&N=4294956287&Nrpp=1000&No=3000&fromPage=plp, anti-MARK3 rabbit polyclonal antibody (ab133708 [Abcam]): https://studylib.net/doc/12744796/anti-mark3-antibody-ab133708-product-datasheet-2-images, anti-phospho-MARK family rabbit polyclonal antibody (#4836 [Cell Signaling Technology]): https://www.cellsignal.jp/products/primary-antibodies/phospho-mark-family-activation-loop-antibody/4836, anti-p38 rabbit monoclonal antibody (#8690 [Cell Signaling Technology]): https://www.cellsignal.jp/products/primary-antibodies/p38-mapk-d13e1-xp-rabbit-mab/8690, anti-phospho-p38 (Thr180/Tyr182) rabbit monoclonal antibody (#4511 [Cell Signaling Technology]): https://www.cellsignal.jp/products/primary-antibodies/phospho-p38-mapk-thr180-tyr182-d3f9-xp-rabbit-mab/4511, anti-p53 rabbit monoclonal antibody (#2527 [Cell Signaling Technology]): https://www.cellsignal.jp/products/primary-antibodies/p53-7f5-rabbit-mab/2527, anti-p70 S6 rabbit monoclonal antibody (#2708 [Cell Signaling Technology]): https://www.cellsignal.jp/products/primary-antibodies/p70-s6-kinase-49d7-rabbit-mab/2708, anti-phospho-p70 S6 (Thr389) rabbit monoclonal antibody (#9234 [Cell Signaling Technology]): https://www.cellsignal.jp/products/primary-antibodies/phospho-p70-s6-kinase-thr389-108d2-rabbit-mab/9234, anti-PAX8 rabbit polyclonal antibody (10336-1-AP [Proteintech, Rosemont, IL, USA]): https://www.ptglab.co.jp/products/PAX8-Antibody-10336-1-AP.htm, anti-YAP rabbit monoclonal antibody (#14074 [Cell Signaling Technology]); https://www.cellsignal.jp/products/primary-antibodies/yap-d8h1x-xp-rabbit-mab/14074, anti-phospho-YAP (Ser127) family rabbit polyclonal antibody (#13008 [Cell Signaling Technology]): https://www.cellsignal.jp/products/primary-antibodies/phospho-yap-ser127-d9w2i-rabbit-mab/13008, Anti-mouse IgG, horseradish peroxidase (HRP)-linked species-specific whole antibody (from sheep) (NA931 [GE Healthcare]): https://www.citeab.com/antibodies/3288287-na931-1ml-amersham-ecl-mouse-igg-hrp-linked-whole-a, anti-rabbit IgG, HRP-linked species-specific whole antibody (from donkey) (NA934 [GE Healthcare]): https://www.citeab.com/antibodies/3288289-na934-1ml-amersham-ecl-rabbit-igg-hrp-linked-whole, anti-mouse IgG (H+L) Alexa Fluor 488 (from donkey) (A21202 [Thermo Fisher Scientific]): https://www.thermofisher.com/antibody/product/Donkey-anti-Mouse-IgG-H-L-Highly-Cross-Adsorbed-Secondary-Antibody-Polyclonal/A-21202, EnVision+ System- HRP Labelled Polymer Anti-Rabbit (K4003 [Agilent Dako]: https://www.agilent.com/cs/library/packageinsert/public/107102005.PDF, biotinylated anti-rat IgG (H+L) (BA-9400 [VECTOR LABLATORIES]): https://vectorlabs.com/biotinylated-goat-anti-rat-igg-antibody.html.

Eukaryotic cell lines

Policy information about cell lines

Cell line source(s)	The 293T (expressing SV40 T-antigen), CaOV3, and ES2 cell lines were purchased from the American Type Culture Collection (ATCC, Manassas, VA, USA). JHOS-2, JHOS-4, and OVCAR3 cells were purchased from the RIKEN CELL BANK (Tsukuba, Japan). KURAMOCHI, OVSAHO, RMUGS, and TYK-nu cell lines were purchased from the Japanese Collection of Research Bioresources Cell Bank (JCRB, Osaka, Japan). 293T and CaOV3 cells were cultured in DMEM with 10% fetal bovine serum (FBS). JHOS-2 and JHOS-4 cells were cultured in DMEM/HamF12 medium with 10% FBS and 0.1 mM NEAA. ES2, KURAMOCHI, and OVSAHO cells were cultured in RPMI1640 medium with 10% FBS, and OVCAR3 cells were maintained in RPMI 1640 medium with 20% FBS and 0.1% insulin. RMUGS cells were cultured in Ham's F12 medium with 10% FBS. TYK-nu cells were cultured in EMEM with 10% FBS.
Authentication	All cell lines were certified by STR profiling cell line authentication (Supplementary Data 4).
Mycoplasma contamination	We routinely confirmed that these cell lines were negative for mycoplasma contamination using an e-Myco mycoplasma PCR detection kit (25235; iNtRON Biotechnology, Kirkland, WA, USA).
Commonly misidentified lines (See ICLAC register)	n/a

Animals and other organisms

Policy information about studies involving animals; ARRIVE guidelines recommended for reporting animal research

Laboratory animals	Mouse xenograft experiments were performed in 8-week-old female BABL/cAJc1-nu/nu mice (CLEA Japan Inc., Tokyo, Japan). Based on weight measurements before injection, the mice were divided into two groups of five mice each so that the mean weight of each group such as DOX positive (+) or DOX negative (-) was approximately equal. MARK3 DOX-inducible OVCAR3 cells were precultured in a medium with 0.2 μg/mL DOX (D9891; Sigma-Aldrich) or the same volume of water, followed by subcutaneous injection of 1×107 cells in the left inguinal areas of mice. The DOX (+) group and DOX (-) control group mice were fed 5% sucrose liquid, containing 2 mg/mL DOX or the same volume of water, respectively. Tumors were resected at 50 days after subcutaneous injection. For CD31 immunostaining to evaluate early phase angiogenesis, additional mouse xenograft experiments were performed, and tumors were resected and paraffin-embedded at 30 days after subcutaneous injection. For tumor diameter measurement, the long and short diameters of the tumor masses were measured by the caliper, and the estimated tumor volume was calculated using the formula as follows:<br>tV = a × b*2 × 0.5 (tumor volume: tV, long diameter: a, short diameter: b)<br>The unit of tumor volume is mm3; the unit for the long (a) and short (b) diameters of the tumor mass was mm, which was measured and calculated to one decimal place. For each of the five mice in the DOX (+) and DOX (-) groups, the mean tumor volume in the tumor mass was calculated, the unpaired Student’s t-test by two-tailed distribution was used for the comparison of two groups.<br>Paraffin sections of the excised tissues were subjected to tissue immunohistochemistry using anti-CD31 antibody, and the CD31 staining area was calculated using the BZ-X800 Analyzer software. Microscopically, the tumors often formed two or three independent masses separated by stroma, rather than a single mass. Therefore, in order to accurately calculate the CD31 staining area within the tumor tissue with the analysis software, each mass had to be cropped on the image data and the CD31 staining area had to be calculated individually. As a result, multiple masses were measured from a single slide, so we calculated the mean value for each slide and performed an unpaired Student’s t-test by two-tailed distribution for the comparison of the two groups: DOX (+) group and DOX (-) group.
Wild animals	n/a
Field-collected samples	n/a
Ethics oversight	This study was approved by the Animal Experiment Committee of UNITECH (approval ID: AGR KGC-180216D-20). Mouse xenograft assays were conducted in accordance with the regulations of the Act on Welfare and Management of Animals, the Standards for the Care and Keeping of Laboratory Animals and Reduction of Pain, the Basic Guidelines of the MEXT, the Guidelines for Appropriate Animal Experimentation, and the Guidelines for Animal Disposal Methods.

Note that full information on the approval of the study protocol must also be provided in the manuscript.
